# Functional mitochondrial respiration is essential for glioblastoma tumour growth

**DOI:** 10.1038/s41388-025-03429-6

**Published:** 2025-05-05

**Authors:** Petra Brisudova, Dana Stojanovic, Jaromir Novak, Zuzana Nahacka, Gabriela Lopes Oliveira, Ondrej Vanatko, Sarka Dvorakova, Berwini Endaya, Jaroslav Truksa, Monika Kubiskova, Alice Foltynova, Daniel Jirak, Natalia Jirat-Ziolkowska, Lukas Kucera, Karel Chalupsky, Krystof Klima, Jan Prochazka, Radislav Sedlacek, Francesco Mengarelli, Patrick Orlando, Luca Tiano, Paulo J. Oliveira, Carole Grasso, Michael V. Berridge, Renata Zobalova, Miroslava Anderova, Jiri Neuzil

**Affiliations:** 1 Institute of Biotechnology, Czech Academy of Sciences, 252 50 Vestec, Czech Republic; 2 Faculty of Science, Charles University, 128 00 Prague 2, Czech Republic; 3 CNC-UC, Center for Neuroscience and Cell Biology, University of Coimbra, 3060-197 Cantanhede, Portugal; 4 CCIBB, Centre for Innovative Biomedicine and Biotechnology, University of Coimbra, 3060-197 Cantanhede, Portugal; 5 PPDBEB, Institute for Interdisciplinary Research, Doctoral Programme in Experimental Biology and Biomedicine, University of Coimbra, 3060-197 Cantanhede, Portugal; 6 Institute of Experimental Medicine, Czech Academy of Sciences, 142 00 Prague 4, Czech Republic; 7 Second Faculty of Medicine, Charles University, 150 06 Prague 5, Czech Republic; 8 Institute of Clinical and Experimental Medicine, 140 21 Prague 4, Czech Republic; 9 Institute of Biophysics and Informatics, First Faculty of Medicine, Charles University, 121 08 Prague 4, Czech Republic; 10 Czech Centre for Phenogenomics, Institute of Molecular Genetics, Czech Academy of Sciences, 142 20 Prague 4, Czech Republic; 11 Department of Life and Environmental Sciences, Polytechnic University of Marche, 60131 Ancona, Italy; 12 Malaghan Institute of Medical Research, Wellington, 6242 New Zealand; 13 First Faculty of Medicine, Charles University, 121 08 Prague 2, Czech Republic; 14 School of Pharmacy and Medical Science, Griffith University, Southport, QLD 4222 Australia

**Keywords:** CNS cancer, Cell growth

## Abstract

Horizontal transfer of mitochondria from the tumour microenvironment to cancer cells to support proliferation and enhance tumour progression has been shown for various types of cancer in recent years. Glioblastoma, the most aggressive adult brain tumour, has proven to be no exception when it comes to dynamic intercellular mitochondrial movement, as shown in this study using an orthotopic tumour model of respiration-deficient glioblastoma cells. Although confirmed mitochondrial transfer was shown to facilitate tumour progression in glioblastoma, we decided to investigate whether the related electron transport chain recovery is necessary for tumour formation in the brain. Based on experiments using time-resolved analysis of tumour formation by glioblastoma cells depleted of their mitochondrial DNA, we conclude that functional mitochondrial respiration is essential for glioblastoma growth in vivo, because it is needed to support coenzyme Q redox cycling for de novo pyrimidine biosynthesis controlled by respiration-linked dihydroorotate dehydrogenase enzyme activity. We also demonstrate here that astrocytes are key mitochondrial donors in this model.

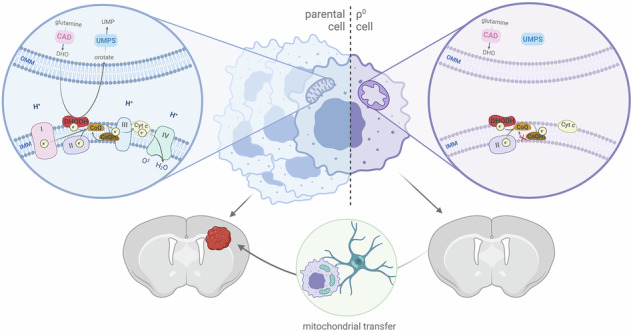

## Introduction

Mitochondria, vital semiautonomous organelles, are essential for important metabolic pathways in the cell, including generation of ATP via oxidative phosphorylation (OXPHOS), as well as for apoptosis or autophagy [1–4]. OXPHOS links mitochondrial respiration involving four respiratory complexes (CI-CIV) of the electron transport chain (ETC) to energy production by phosphorylation of ADP at complex V (CV). Several subunits of respiratory complexes, with the exception of CII, are encoded by mitochondrial DNA (mtDNA) present in the mitochondrial matrix [5]. Moreover, mitochondrial metabolism integrates several biosynthetic pathways important for cell growth and proliferation, such as biosynthesis of nucleic acid building blocks, purines and pyrimidines [6, 7]. Cancer cells that are in constant need of these metabolites for rapid proliferation cannot rely on salvage pathways for pyrimidine replenishment [8, 9].

A protein with a crucial role in the de novo pyrimidine biosynthetic pathway, dihydroorotate dehydrogenase (DHODH), located at the inner mitochondrial membrane, requires coenzyme Q (CoQ) redox cycling for function. It catalyses conversion of dihydroorotate (DHO) to orotate with the resulting electrons generated feeding into the ETC [10]. CoQ redox cycling, in turn, is driven by functional ETC components, CIII and CIV, that intercept electrons from the reduced form of CoQ, ubiquinol (UbQH_2_), which is then reoxidised to ubiquinone (UbQ) [11, 12]. This series of reactions links de novo pyrimidine biosynthesis to functional ETC via DHODH. We have shown impaired growth of tumours from DHODH-deficient melanoma and breast cancer cells in mouse models, while others have shown impaired tumour growth for CIII-deficient cells in orthotopic lung cancer and acute lymphoblastic leukaemia models [13, 14]. Recent publications, therefore, suggest that ATP production via OXPHOS, is dispensable for tumour growth, while functional ETC is essential [14–16].

Glioblastoma (GBM) is the most frequently occurring and aggressive primary brain tumour. Patients diagnosed with GBM face a very poor prognosis, with 5-year survival of less than 10% and a median overall survival of 14 months after diagnosis [17, 18]. The current standard treatment for GBM consists of surgical resection followed by radiation therapy and chemotherapy. However, complete resection is almost impossible due to the diffuse character of GBM in the brain, and radiotherapy is inefficient in hypoxic areas of the tumour [19], highlighting the importance of innovative treatment approaches. Exploring the novel concept of horizontal mitochondrial transfer (HMT) in glioblastoma offers the potential to uncover new mechanisms of cellular communication and energy transfer, which could not only enhance our understanding of tumor biology but also lead to the development of more effective therapeutic strategies aimed at disrupting these processes and improving patient outcomes.

We have previously reported HMT in vivo using a models of respiration-deficient ρ^0^ tumour cell lines. These cells, devoid of mtDNA that encodes several subunits of respiratory complexes, lacked tumour-forming ability in mouse models unless they acquire mitochondria with mtDNA from the host [20–22]. The occurrence of HMT has now been reported in various pathological conditions, including cancer, as well as for physiological processes such as wound healing [23–25]. In the brain, HMT has been studied primarily in neurodegenerative diseases where it has been shown to occur between stressed and healthy astrocytes in Parkinson’s disease, and between astrocytes and neurons in Alzheimer’s disease [26, 27]. Moreover, a recent study reported that HMT in glioblastoma resulted in increased mitochondrial respiration, ATP production, and tumour growth [28]. As astrocytes, the predominant glial cell type in the brain, have been proposed as mitochondrial donors [29, 30], we also explored the origin of transferred mitochondria in our model system both in vitro and in vivo.

To establish the importance of functional ETC in the context of brain cancer growth, we used an orthotopic mouse model of glioblastoma to preserve the authentic GBM microenvironment. Using a model of ρ^0^ GBM cells with compromised ETC, we observed that tumours do not form unless the implanted ρ^0^ cells obtain respiration-competent mitochondria from cells in the host microenvironment, indicating that a functional ETC is indispensable for tumour growth in the brain. This is in line with recent observations that functional ETC is necessary for the growth of various tumours, although this has not been previously shown for brain cancer [14, 31, 32]. Furthermore, our study identified astrocytes as being the most prominent mitochondrial donor cell type within the brain. In general, our results suggest that therapeutic interventions targeting DHODH enzyme activity or based on inhibition of HMT could potentially increase the effectiveness of glioblastoma treatments.

## Methods

### Cell culture

GL261 murine glioblastoma cell line was purchased from the ATCC and aliquots were frozen and kept in liquid nitrogen. GL261 ρ^0^ cell line was prepared from the parental cell line by long-term (10–12 weeks) cultivation in medium with low-dose ethidium bromide (EtBr, 50–100 ng/mL). The loss of mtDNA was monitored by quantitative PCR, and respiration deficiency was confirmed by Seahorse and Oxygraph analyses. Unless stated otherwise, all cell lines were maintained in DMEM high glucose medium (Sigma-Aldrich) with 10% FBS (Sigma-Aldrich), 1% penicillin/streptomycin (Sigma-Aldrich) and supplemented with additional 1 mM sodium pyruvate (Sigma-Aldrich), 50 μg/mL uridine (Sigma-Aldrich) and 5 mg/mL glucose (Sigma-Aldrich). All cell lines were routinely tested for mycoplasma contamination.

### Lentiviral transduction

GL261 parental and ρ^0^ cells were transduced with lentiviral particles bearing the GFP or the mito:mKate2 plasmid (Addgene). Viral particles were obtained by precipitation with PEG-it (System Biosciences) from the medium 48 h post-transfection of HEK 293 T cells with two packaging psPAX2 and pMD2.G plasmids (both from Addgene, gift from Didier Trono, #12260 and #12259 respectively) together with the pKAM-GFP plasmid (Addgene, gift from Archibald Perkins, #101865). Cells were selected based on the presence of the fluorescent signal by fluorescence-activated cell sorting (FACS).

### CRISPR/Cas9 knock-out generation

GL261 cells were transfected using a 100 μL tip electroporation system (Neon, Thermo Fisher Scientific). 1.2 × 10^6^ cells were harvested and centrifuged (1200 rpm, 5 min). The cell pellet was resuspended in 120 μL of R (re-suspension) buffer, and 5 μg of plasmid targeting DHODH gene (the guide RNA sequences used: 5’-TCA GGT ACT CGG CGT AGA AA-3’) was added to cells. Electroporation conditions used for the transfection were as follows: pulse voltage - 1200 V; pulse width -20 ms; pulse number -2. After transfection, cells were seeded in antibiotic-free media and cultivated for 2-3 days. Transfected cells were selected by puromycin (2 μg/mL) for 5 days. Surviving cells were sorted as single cells into 96-well plates, cultivated in media supplemented with uridine and pyruvate, expanded and tested for the absence of DHODH protein by western blotting (WB).

### Mouse models

All in vivo orthotopic tumour experiments were performed using 10–15 week-old C57Bl/6 mice, syngeneic for GL261 cells. GL261-GFP, GL261 ρ^0^-GFP, D30, D75, D106 and GL261 DHODH KO cells were injected intracranially into the right cerebral hemisphere using stereotaxic coordinates 2 mm caudal and 2 mm lateral to the coronal and sagittal sutures, respectively, 2.5 mm deep into the brain parenchyma. 5 × 10^4^ cells in 3 μl of PBS per animal were implanted. To ensure cell viability during the time-consuming intracranial injection procedure, 8–10 animals were injected per procedure. Therefore the sample sizes reflect this limitation. Only animals with tumour at the endpoint of the experiment were included in the analysis of in vivo experiments. For in vivo pharmacological inhibition of DHODH, mice were injected with 5 × 10^4^ GL261-GFP cells as described and randomly divided into the control group (*n* = 5) and the treated group (*n* = 4) by the investigators. Control mice were given vehicle solution (10% DMSO in corn oil) by daily gavage, while treated mice were given 3 mg/kg BAY-2402234 solution in DMSO and corn oil daily by gavage. The investigators were not blinded to the group allocation at any stage of the experiment. Tumour presence was estimated based on weight loss of the animals, with 15% weight loss plus behavioural changes indicative of tumour presence. This was considered the ethical endpoint of the experiment. The behavioural changes included hunched posture and reduced exploration. The weight loss percentage was calculated relative to the highest weight the animal has achieved thoughout the experiment. Tg(CAG-mKate2)1Poche/J (mito::mKate2; JAX no. #032188) mice expressing the mKate2 fluorophore in mitochondria were used to isolate neural cell types for in vitro co-culture experiments.

For in vivo HMT monitoring experiments, the Aldh1l1-creERT2 [B6N.FVB-Tg(Aldh1l1-cre/ERT2)1Khakh/J; JAX no. #031008] and the MitoTag [B6N.Cg-Gt(ROSA)26Sortm1(CAG-EGFP*)Thm/J; JAX no. #032675] mouse strains were obtained from The Jackson Laboratory and crossed to generate Aldh1l1:MitoTag experimental animals. In these mice, a tamoxifen-induced cre-mediated DNA recombination results in the expression of an enhanced green fluorescent protein (EGFP) targeted into the outer mitochondrial membrane in astrocytes (Aldh1l1 promoter). Cre-mediated DNA recombination was achieved by five intraperitoneal injections of tamoxifen (100 mg/kg of body weight; Toronto Research Chemicals) dissolved in corn oil (Sigma-Aldrich) for 5 consecutive days, starting at P42 ± 2 days. 10–14 days after the last tamoxifen injection, the animals were implanted with 5 × 10^4^ GL261 ρ^0^-mito:mKate2 cells using the following coordinates: [AP: 0 mm, ML: 2 mm, DV: 3 mm] and the brains were isolated after 6 weeks for microscopy analysis.

Unless stated otherwise, mice of both sexes were used for the experiments. The mice were housed in standard breeding cages under controlled conditions (22 ± 2 °C and relative humidity 30 ± 5%), on a 12:12 dark/light cycle. Food and water were provided *ad libitum*. Enrichment was provided in the form of paper towels. All procedures involving the use of laboratory mice were performed in accordance with the European Communities Council Directive of November 24, 1986 (86/609/EEC) and the Animal Welfare Guidelines. All procedures were approved by the Animal Care Committee of the Czech Academy of Sciences.

### Magnetic resonance imaging (MRI)

All animal procedures were conducted in accordance with ethical guidelines and approved by the Ethics Committee of the Institute for Clinical and Experimental Medicine and the Ministry of Health of the Czech Republic (Approval No. 58/2014). The protocols adhered to the European Communities Council Directive (2010/63/EU) on the protection of animals used for scientific purposes. During the magnetic resonance imaging (MRI) experiments, anesthesia was induced using 5% isoflurance and maintained at 1.5 to 2% (Baxter). Respiratory function was continuously monitored using a trigger unit (Rapid Biomedical), and eye protection was ensured by applying eye cream (Ophthalmo-Septonex, Zentiva). The animals were placed in a custom coil holder equipped with water-filled tubes to maintain body temperature at 37 °C during MR measurements. Hydrogen MRI (^1^H-MRI) was performed using a BioSpec 47/20 preclinical 4.7 T scanner (Bruker) with a custom-made half-saddle surface coil (30 mm diameter) placed over the animal’s head. Tumor imaging was conducted using a *T*_*2*_-weighted turbo spin-echo sequence (Rapid Acquisition with Relaxation Enhancement, RARE) in both axial (27 slices) and coronal (15 slices) planes. The imaging parameters were as follows: repetition time (TR) = 3300 ms, effective echo time (TE) = 36 ms, turbo factor = 8, 4 acquisitions (total scan time 5 min 16 s), field of view (FOV) = 3.5 × 3.5 cm, matrix size = 256 × 256, slice thickness = 0.6 cm, voxel resolution = 0.0112 µL. Imaging was performed at predetermined time points following cell transplantation (once every 2 weeks for the first 2 months, then once every week), starting on day 7. Tumor growth over time was assessed through quantitative analyses of ^1^H-MRI data. Volumetry was determined by manually marking the tumor area using ImageJ software (version 1.46r, National Institutes of Health, USA). To enhance the outcome precision, lesion size was calculated as the mean of the segmented axial and coronal tumor areas from the MR images.

### Isolation of tumour-derived cell lines

Cell lines were established from mice on days 30, 75 and 106 from the primary orthotopic tumours/pre-tumour sites, after grafting 5 × 10^4^ GL261 ρ^0^ GFP cells into the brain of C57Bl/6 mice. The time points were chosen to reflect different stages of tumour growth, with D30 cells isolated from the pre-tumour site not detectable by MRI, D75 cells isolated from a small tumour (4.08 mm^3^) just detectable by MRI but without animal weight loss, and D106 cells derived from a large tumour (approx. 85 mm^3^) at the ethical endpoint (animal weight loss, behavioural changes). Tumour (D106) or broader tumour injection site (D30, D75) tissues were excised from the brain of sacrificed mice under sterile conditions, cut into several pieces and placed in media until cells attached to the culture dish. Tissue pieces were removed, and attached cells were passaged. Tumour cells outgrew other types of brain cells and were selected by FACS based on the presence of GFP fluorescence and maintained as stable cell lines.

### mtDNA level assay

Total DNA was extracted in triplicate from individual cell lines, isolated brain cells and GL261 ρ^0^-GFP cells co-cultured with neural cells for 72 h using the Wizard Genomic DNA Purification Kit (Promega) according to the manufacturer’s protocol. DNA purity and concentration were assessed using NanoDrop (ThermoFisher Scientific). 190 ng of DNA was combined with forward and reverse primers for *Mito1* (mtDNA) and *Rn18s* (nDNA; primer sequences listed in Supplementary Table 1) and HOT FIREPol EvaGreen qPCR Supermix (Solis Biodyne). The reaction was run on CFX384 Touch Real-Time PCR Detection System (Bio-Rad) with the following settings: initial denaturation (95 °C for 12 min) followed by 38 cycles of denaturation, annealing and extension (95 °C for 15 s, 60 °C for 20 s and 72 °C for 20 s, respectively). The mtDNA level was determined relative to the nuclear-coded *Rn18s* gene using the ΔΔC_t_ method.

### mtDNA sequencing

Total DNA containing mtDNA was isolated from cell pellets using the DNAzol reagent (Molecular Research Center) according to manufacturer´s instructions. Full-length mtDNA was sequenced by amplifying ten 1.8 kb dsDNA PCR products using the 5x HOT FIREPol Blend Master Mix with 10 mM MgCl_2_ (primer sequences listed in Supplementary Table 1). PCR products were separated on agarose gels and purified using the DNA clean-and-concentrator kit (Zymoresearch). Purified PCR products (125 ng) were combined with water and 1 µL of 25 µM primer stocks to yield 10 µL sequencing reaction mixture. Sanger sequencing was performed via Euroffin Genomics Light Run service. Sequences were aligned with murine C57Bl/6 strain mtDNA using the SeqManPro suite from the DNASTAR Lasergene 17 software enabling visualisation of the peak histograms.

### Quantitative real-time (reverse transcription) PCR

Total RNA was isolated from individual cell lines (seeded in 6-well plates at the density of 2 x 10^5^ cells per well and cultivated for 24 h) or from tissue homogenates (using Precellys homogenisation tubes, Bertin Technologies) by means of RNAzol extraction (Molecular Research Center), and its purity and concentration was measured by NanoDrop (ThermoFisher Scientific). 1 μg of RNA was used for reverse transcription into cDNA using RevertAid RT Reverse Transcription Kit (ThermoFisher Scientific) with random hexamer primer. To assess the expression of target genes (primer sequences listed in Supplementary Table 1), 250 ng of cDNA was used for each qRT-PCR reaction containing HOT FIREPol EvaGreen qPCR Supermix (Solis Biodyne). qRT-PCR was performed using the CFX384 Touch Real-Time PCR Detection System (Bio-Rad) with the following settings: initial denaturation (95 °C for 12 min) followed by 38 cycles of denaturation, annealing and extension (95 °C for 15 s, 60 °C for 20 s and 72 °C for 20 s, respectively). Target genes were normalised to the housekeeping gene *Actb*, and change in gene expression was determined using the ΔΔC_t_ method.

### Immunohistofluorescence

Brains were excised after transcardial perfusion with PBS followed by perfusion with 4% paraformaldehyde (PFA) and incubated in 4% PFA for 24 h at 4 °C. Tissue was washed with PBS, incubated in DMSO for 1 h, incubated in increasing concentrations of OCT embedding matrix (VWR chemicals), mounted in 100% OCT in cryomolds and frozen at −80 °C. Tissue was then sectioned using a cryotome (Leica) into 5 μm thick slices, mounted on glass slides and stored at −20 °C. The staining procedure was carried out as follows: slides were soaked in PBS, permeabilised with saponin, blocked with 10% goat serum in 0.1% Tween-20 and incubated with primary antibodies (Supplementary Table 2) overnight, washed 3 x with PBS and incubated with respective fluorescent secondary antibodies (Supplementary Table 2) for 2 h in the dark. Samples were counterstained with DAPI (Invitrogen) and mounted in Vectashield Vibrance antifade mounting medium (Vector Laboratories).

For in vivo HMT monitoring experiments, the mice were deeply anesthetized with pentobarbital (i.p., 100 mg/kg, in saline) and transcardially perfused with cold saline (with 30 IU/ml heparin) followed by perfusion with 4% PFA (in 0.1 M PBS). The brains were isolated and postfixed 3 h with PFA, and treated with a 10/20/30% sucrose gradient for 16/24/48 h, respectively. The brains were then cut with a Leica CM1850 cryostat into 30 μm coronal slices. The slices were washed in PBS and incubated for 2 h in a blocking solution (5% Chemiblocker and 0.5% Triton X-100 in 10 mM PBS). The slices were then incubated with antibodies and counterstained as described above and mounted on microscopy slides using Aqua Poly/Mount.

The images were acquired using the Nikon Eclipse Ti2 confocal laser scanning microscope fitted with spinning disk module Yokogawa CSU-W1 (Nikon) or by Zeiss LSM 880 confocal laser scanning microscope (Carl Zeiss Microscopy), and processed in FIJI software (ImageJ 2.9.0/1.53t; 10.1038/nmeth.2019).

### Mitochondrial network visualisation by confocal microscopy

For the visualisation of mitochondrial network, cells were grown in 35 mm glass bottom Petri dishes (Cellvis) for 24 h. Mitochondria were labelled with 100 nM MitoTracker™ Deep Red (Thermo Fisher Scientific) for 30 min in the dark, washed with PBS, and the culture medium was replenished. Cells were imaged under standard culture conditions on a TCS SP8 WLL SMD-FLIM microscope (Leica) and processed by LasX software (Leica).

### Stimulated emission depletion (STED) microscopy

To visualise the presence of DNA in mitochondria, cells were grown on high-performance coverslips in 6-well plates overnight and fixed with 4% paraformaldehyde for 15 min at room temperature. Samples were then incubated in permeabilisation solution (0.1 M glycine, 0.05% TritonX-100, 0.05% Tween-20 in PBS) for 15 min and blocked with 10% BSA for 30 min. Samples were stained overnight with primary antibodies against DNA (Progene, 1:150) and Tomm20 (Abcam, 1:100) in staining solution (1.5% BSA, 0.05% Saponin, 0.01 M glycine in PBS). This was followed by staining with appropriate secondary antibodies Abberior STAR RED (anti-mouse, 1:500) and Abberior STAR 580 (anti-rabbit, 1:500) for 2 h in the dark. Coverslips were mounted in the Vectashield Vibrance antifade mounting medium and kept at 4 °C. STED images were acquired on an Inverted confocal microscope Nikon Eclipse Ti-E equipped with Nikon CFI Plan Apo Lambda objective (60 x Oil, NA 1.40) and STED depletion laser (775 nm, 40 MHz pulsed laser, 2D doughnut) and processed using the FIJI software (ImageJ 2.9.0/1.53t; 10.1038/nmeth.2019), with additional signal deconvolution in Huygens software (Scientific Volume Imaging).

### Transmission electron microscopy (TEM)

Cells were harvested and embedded in 2% low-melting agarose. After fixation with 2% formaldehyde and 2.5% glutaraldehyde in 0.1 M PHEM buffer, samples were postfixed and contrasted for 1 h with 1% osmium tetroxide and 1.5% potassium hexacyanoferrate (III), and for 30 min with 1% uranyl acetate. Dehydration by series of diluted ethanol solutions (30%, 50%, 70%, 80%, 90% and 100%) was followed by acetone incubation and embedding in Epon epoxy resin for cutting 70 nm sections using the Leica EM UC7 Ultramicrotome, and imaged with the JEOL JEM 2100-Plus 200 kV transmission electron microscope. For each sample, at least ten different fields of view (FOVs) were captured and analysed manually in FIJI software (ImageJ 2.9.0/1.53t; 10.1038/nmeth.2019).

### Mitochondrial membrane potential assay

To assess mitochondrial membrane potential by flow cytometry, cells were seeded 24 h before the assay in 6-well plates in triplicates to obtain the following samples: unstained control, TMRM staining and FCCP treatment + TMRM staining. For the mitochondrial membrane potential dissipation, FCCP (10–20 μM) was added directly to the respective wells 5 min before adding TMRM (50 nM) for 15 min at 37 °C. Cells were harvested by trypsin/EDTA and resuspended in cold flow cytometry buffer (PBS, 1% BSA, 1% azide). To exclude dead cells from the analysis, 4 ng/ml Hoechst 33258 was added before measurement with BD LSRFortessaTM SORP and obtained data were assessed by FlowJo software (BD Biosciences, v10.1.0). TMRM signal was normalised to the mitochondrial amount (MitoTracker™ Deep Red signal measured by flow cytometry).

### Seahorse assay

Oxygen consumption rate (OCR) and extracellular acidification rate (ECAR) evaluation were performed using the Seahorse XFe 96 analyser (Agilent Technologies). 24 h before the experiment, cells at 2 x 10^4^ per well were seeded in Seahorse XF96 cell culture microplates in the cultivation medium. The following day, cells were washed with the Seahorse base medium and incubated at 37 °C for 1 h. The Seahorse base medium (180 μL/well) was supplemented with 500 mM pyruvate, 2 mM L-glutamine, and 10 mM glucose for OCR measurements or with 2 mM L-glutamine for ECAR measurements. The Mito Stress Test was run employing a series of additions, starting with oligomycin (1 μM, inhibitor of the ATP synthase, port A), followed by carbonyl cyanide 4-(trifluoromethoxy) phenylhydrazone (FCCP, 2 μM, mitochondrial uncoupler, port B), and a combination of antimycin and rotenone (0.5 μM, inhibitors of CIII and CI, port C). The Glycolytic Stress Test was performed by injection of glucose (10 mM, substrate for glycolysis, port A), followed by oligomycin (1 μM, port B) and 2-deoxy-D-glucose (2-DG, 50 mM, glycolysis inhibitor, port C). OCR and ECAR readings were taken after each injection. Results were normalised to the cell number assessed by the Cytation 5 Cell Imaging Multi-Mode Reader (BioTek) at the end of the experiment.

### Oxygraph measurement

Oxygraph-2k respirometer (Oroboros Instruments) was used to evaluate CI- and CII-dependent respiration of homogenised tissues. Freshly excised tumour and healthy contralateral brain tissue was weighed and homogenised using the PBI-Shredder (Oroboros Instruments), resuspended in the G-medium (0.5 mM EGTA, 3 mM MgCl2, 60 mM K-lactobionate, 20 mM taurine, 10 mM KH_2_PO_4_, 110 mM sucrose, 1 g/L essentially fatty acid-free bovine serum albumin, 20 mM Hepes, pH 7.1) and placed into the instrument chamber maintained at 37 °C. CI-dependent respiration was evaluated in the presence of 10 mM malonate (CII inhibitor) after the addition of 15 mM glutamate, 3 mM malate, 3 mM ADP and 10 µM cytochrome c, while CII-dependent respiration was assessed in the presence of 0.5 µM rotenone (CI inhibitor) after the addition of 10 mM succinate, 3 mM ADP, and 10 μM cytochrome c into the chamber. Residual oxygen consumption after inhibition with 2.5 μM antimycin A (CIII inhibitor) was subtracted from the results. Data were normalised to tissue weight to obtain specific oxygen consumption rates. DHODH-dependent respiration was evaluated as follows: cells were harvested with trypsin/EDTA, resuspended in the G-medium and placed into the instrument chamber maintained at 37 °C. Cells were permeabilised with digitonin (5 μg/10^6^ cells), followed by sequential addition of 0.5 µM rotenone, 1 mM dihydroorotate (DHO), 3 mM ADP, 10 μM cytochrome c, 30 µM teriflunomide (DHODH inhibitor) and 2.5 μM antimycin A. DHODH-dependent respiration was calculated from values after the addition of cytochrome c and substraction of residual oxygen consumption following addition of teriflunomide and antimycin A and normalised to the cell number.

### ATP quantification

ATP level was evaluated using the Cell-Titer Glo kit (Promega) according to the manufacturer’s instructions with 2-DG used to differentiate between ATP formed by OXPHOS and total ATP. Briefly, cells were seeded in 96-well plates at 5 x 10^3^ per well. After 24 h, cell culture media was changed with either control or 2-DG (50 mM) containing media, and cells were incubated for another 24 h. 100 µL of Cell-Titer Glo mixture was added to each well and incubated for 10 min at RT, and luminescence was assessed using the Infinite M200 microplate reader (Tecan). Data were normalised to protein concentration assessed by the bicinchoninic acid (BCA) assay.

### Mitochondrial superoxide anion analysis

Mitochondrial superoxide anion levels were evaluated by flow cytometry using the MitoSOX probe. Cells were seeded 24 h before the experiment, harvested by trypsin/EDTA detachment and washed once with culture media. The cells were then incubated with 3 μM of MitoSOX for 30 min at 37 °C in a shaking heating block, protected from light. Following incubation, cells were washed three times with warm PBS, and resuspended in 200 ul of PBS. To exclude dead cells from the analysis, 1 μg/ml Hoechst 33258 was added before evaluation using BD LSRFortessaTM SORP, and the obtained data were assessed by the FlowJo software.

### Western blotting

Cells were lysed, and tissues were lysed and homogenised (using Precellys homogenisation tubes (Bertin Technologies) in the RIPA lysis buffer (20 mM Tris-HCl, pH 7.4, 1 mM EDTA, 150 mM NaCl, 1% Triton X-100, 0.1% SDS, 0.5% sodium deoxycholate) supplemented with protease inhibitor cocktail (1:100, Thermo Fisher Scientific). Protein concentration was measured using the BCA assay, and 30–50 μg of protein per sample was loaded onto a sodium dodecyl sulfate-polyacrylamide gel (SDS-PAGE). After transfer to nitrocellulose membranes (BioRad), separated proteins were probed with selected primary antibodies (listed in Supplementary Table 2), followed by goat anti-rabbit IgG (H + L) HRP conjugate or goat anti-mouse IgG (H + L) HRP conjugate (both BioRad) secondary antibodies (dilution 1:10 000). Images were acquired using the Azure c600 imaging system (Azure Biosystems), and signals from 3 biological replicates were quantified by densitometry relative to the housekeeping proteins β-actin or GAPDH.

### Isolation of mitochondria

Harvested cell pellets were resuspended in the STE buffer (250 mM sucrose, 10 mM Tris pH 7.6, 1 mM EDTA) and homogenised by passing three times through an 8 µm tungsten carbide ball of the Balch-style cell homogeniser (Isobiotec) using a hand-driven 1 mL syringe. Mitochondria were isolated by several rounds of centrifugation at 4 °C (800 *g* for 5 min, 3 000 *g* for 5 min and 10,000 *g* for 15 min). The mitochondrial pellet was washed with STE buffer and stored at −80 °C.

### Blue native gel electrophoresis (BNGE)

Mitochondria were solubilized with digitonin (8 g/g of protein) and 10 μg (for CV and Hsp60) or 20 μg (for CI, CIII and CIV) of mitochondrial protein in the sample loading buffer (0.015 μL/μg protein; 0.75 M aminocaproic acid, 50 mM Bis-Tris, 12% glycerol, 0.5 mM EDTA, 5% Coomassie Brilliant Blue G-250) was separated on 3–12% NativePAGE Novex BisTris gradient gels (Invitrogen). Electrophoresis ran in three steps, i.e., using the blue cathode buffer (0.02% Coomassie Brilliant Blue G-250) at 35 V for 70 min, and then clear cathode buffer at 25 V overnight. Finally, the voltage was increased to 200 V for 2 h. Then, gels were incubated in transfer buffer containing 0.1% SDS for 10 min and proteins were transferred onto 0.2 μm polyvinylidene difluoride (PVDF) membranes (BioRad) probed with primary antibodies against components of respiratory complexes CI (anti-NDUFA9, ab14713), CIII (anti-UQCRC2, ab14745), CIV (anti-COX5A, ab110262) and CV (anti-ATP5β, HPA001520). Anti-HSP60 (#12165) was used as loading control.

### High resolution clear native electrophoresis (hrCNE)

30 μg (for CI) or 50 μg (for CV) of digitonin-solubilised mitochondria were mixed with 10x sample buffer containing 50% glycerol and 0.1% Ponceau S dye and subjected to high-resolution clear native electrophoresis on a 3–12% (for CI) or 4–16% (for CV) NativePAGE Novex BisTris gradient gels (Invitrogen). Cathode buffer was supplemented with 0.05% deoxycholate and 0.01% lauryl maltoside. Electrophoresis was performed at 35 V for 70 min, followed by overnight running at 25 V and an additional 2 h at 200 V. Then, gels were incubated for 10 min in an assay buffer containing 0.01% NADH, 0.25% nitrotetrazolium blue in 5 mM Tris-HCl (pH 7.4) for CI in-gel activity. The CV in-gel activity was evaluated by pre-incubation in 35 mM Tris and 270 mM glycine buffer (pH 8.3) for 2 h followed by incubation in an assay buffer composed of 140 mM MgSO_4_, 0.2% Pb(NO_3_)_2_ and 0.44% ATP in 35 mM Tris and 270 mM glycine buffer (pH 8.3) for 2 h. Gels were visualised after the reaction was terminated by solution of 50% methanol and 10% acetic acid.

### Cell proliferation analysis

Cell proliferation was determined using the IncuCyte SX1 analyser (Sartorius & Essen BioScience). On the day of the experiment, cells were seeded in a 96-well plate (5 x 10^3^ cells/well). The plate was scanned with a 10x objective every 3 h for 96 h, and the obtained results were analysed using the Incucyte Analysis Software Module.

### Isolation and cultivation of mito::mKate2 mouse neural cells

Neural cells were isolated by magnetic separation from adult Tg(CAG-mKate2)1Poche/J (mito::mKate2) mouse brains dissociated using Adult Brain Dissociation Kit, mouse and rat (Miltenyi Biotec) according to the manufacturer’s protocol. Magnetic separation was carried out using LS-columns with anti-ACSA-2 MicroBead Kit (astrocytes), and anti-CD11b MicroBeads (microglia), according to manufacturer’s protocols. After magnetic separation and cell counting, 10^5^ live cells were plated in 50 μL of culture media in poly-L-lysine coated dishes, and the culture medium was replenished 30 min later to allow better initial attachment. Cells were maintained in culture without passaging, and half of the cultivation media was changed every 2–3 days. Astrocytes were cultured in AstroMACS Medium (Miltenyi Biotec) and microglia were maintained in high-glucose DMEM (Sigma-Aldrich) supplemented with 1% antibiotics and 250 nM L-glutamine (PAN-Biotech).

### In vitro co-culture assays

Astrocytes and microglia positive for mito::mKate2 were isolated as described, and 10^5^ cells were seeded in poly-L-lysine coated 35 mm glass bottom Petri dishes (Cellvis) for confocal microscopy or in 6-well plates (Starstedt) for flow cytometry experiments. After 2–3 weeks, 10^5^ GL261 ρ^0^ GFP cells were added to neural cells and cultivated for 24–72 h. To visualise and quantify mitochondrial transfer, co-cultures of cells were subjected either to live-cell confocal imaging (Nikon Eclipse Ti2 microscope with spinning disk module Yokogawa CSU-W1) or harvested into single-cell suspension and assessed by flow cytometry (BD LSRFortessaTM SORP). Data from confocal microscopy were processed using the FIJI software (ImageJ 2.9.0/1.53t; 10.1038/nmeth.2019) and flow cytometry data were analysed using FlowJo software (BD Biosciences, v10.1.0). Mitochondrial transfer was quantified as percentage of double positive (mKate2^+^, GFP^+^) cells of all GFP^+^ live cells after doublet exclusion.

### Stable isotope tracing

Stable isotope tracing was measured upon cell incubation with uniformly-labelled L-glutamine (L-Glutamine-^13^C_5_,^15^N_2_, Sigma-Aldrich 607983). Briefly, 3 × 10^5^ cells were seeded in 60 mm cell culture dish in cultivation medium. After 24 h, cells were thoroughly washed and placed in glutamine-free media (glutamine-free DMEM-F12 medium L0091, BioWest) supplemented with 2 mM labelled L-glutamine for 24 h incubation. After the incubation the cells were pelleted. Frozen cell pellets (7,5 × 10^5^ cells) were mixed with 150 µL of solvent (40% methanol, 40% acetonitrile, 20% water) to precipitate proteins. Samples were sonicated, incubated at −20 °C for 12 h and centrifuged (10 min, 20,000 *g*). Supernatants were collected for analysis by Liquid Chromatography Tandem Mass Spectrometry (LC-MS/MS). LC separation was done using iHILIC®-(P) Classic, PEEK, 100 x 2.1 mm, 5 µm, 200 Å column (Hilicon) using water/acetonitrile gradient with 10 mM ammonium formate, starting from 95% until 42% for 15 min in both polarities. LC-MS/MS analysis was conducted on ID-X Orbitrap (Thermo Fisher Scientific). Xcalibur software (Thermo Fisher Scientific, v 4.3) was used for data acquisition, and Compound Discoverer 3.2 was used for data analysis. Raw data were aligned by retention time, features were merged, and data were grouped into experimental groups. A QC mix sample was used for the identification of metabolites by annotating MS/MS spectra using mzCloud, mzLogic, and a custom mass list database with retention time.

### MALDI imaging

Mice were sacrificed by cervical dislocation and intact brains were carefully extracted and washed in warm 2% and 10% gelatin solution (Dr. Oetker). Brains were flash-frozen in 10% gelatin solution in isopentane bath (Sigma Aldrich) cooled with liquid nitrogen. Samples were stored at −80 °C prior to cryo-sectioning into 10 µm slices using the CM1950 cryostat (Leica Biosystems). Tissue slices were thawed and mounted on warm ITO glass slides (Ossila). Mounted samples were dried in a desiccator for 15 min and stored at −80 °C. Before spraying, slides were tempered to room temperature for 30 min and dried for additional 15 min in a desiccator. Samples were sprayed with 10 mg/ml 9-aminoacridine matrix (9AA, Merck) in 70% ethanol with the following parameters: spray nozzle heated to 50 °C, flow rate 50 µL/min. Slides were sprayed with 12 layers with horizontal nozzle movement at 1000 mm/min, nitrogen flow 2 L/min, gas pressure 10 psi, 2 mm distance between each pass, nozzle distance 42 mm. MALDI-TOF MS spectra were acquired using the rapifleX MALDI-TOF/TOF spectrometer (Bruker) operated by the fleXcontrol software v4.2 (Bruker) in the reflector negative mode with a 355 nm Smartbeam™ 3D laser with spatial resolution of 50 × 50 µm in the m/z range of 20–1000, at constant laser fluence of 88% and laser frequency of 5 kHz. 200 shots were summed up from every position. The instrument was set up as follows: ion source 1, 19.973 kV; PIE, 2.664 kV; lens, 11.353 kV; reflector 1, 20.810 kV; reflector 2, 1.034 kV; reflector 3, 8.577 kV. The pulsed ion extraction time was set to 90 ns and detector gain to 2302 V. Data were acquired with the digitizer speed at 2.5 GS/s. Calibration was done externally using red phosphorus [33] (Kolářová et al. 2017) with precision up to 5 ppm. Spatial navigation for imaging data acquisition was done in the flexImaging v6.0 software (Bruker). After MALDI imaging, the matrix layer was removed from samples with 70% ethanol. Sample was washed with distilled water, subjected to hematoxylin-eosin (H&E) staining, dehydrated with series of ethanol, isopropanol and xylene, and mounted in permanent medium (Pertex, Bamed) in the Leica ST5020 automated staining instrument in combination with the Leica CV5030 coverslipper (Leica Biosystems). Stained tissues were scanned with Axio Scan.Z1 (Zeiss) with 10x objective and processed with the Zen 2 software (blue edition; Zeiss). Data files were imported as raw spectra into SCiLS software (Bruker, ver. 2024b). Images stained with H&E were loaded into the MALDI imaging dataset to enable analysis based on histology. Spectra were normalized using the total ion current method, and images were interpolated and displayed with medium de-noising. Mean intensity of regions of interest were reported.

### Analysis of oxidized and reduced CoQ

Cells were grown in 100 mm culture dishes for 48 h, washed and harvested into cold PBS, pelleted in conical-bottom cryotubes filled with argon and frozen on dry ice. Samples were kept at −80 °C until further analysis. Cell pellets were resuspended in the extraction buffer (1-propanol/ PBS, 5:1), vortexed and centrifuges at 20,900 *g* at 4 °C for 2 min. Oxidised (UbQ) and reduced (UbQH_2_) forms of coenzyme Q_9_ (CoQ_9_; major form of coenzyme Q present in rodents) in the supernatant were evaluated simultaneously by high-performance liquid chromatography (HPLC) with electrochemical detection (ECD) and a post-analytical reducing column using a Nanospace HPLC apparatus (Shiseido, Tokyo, Japan). The samples were loaded with the mobile phase 1, which consisted of 50 mM sodium perchlorate in methanol/distilled water (95/5, v/v) with the flow rate of 200 μL/min. The samples were subsequently eluted from the concentrating column by mobile phase 2, which consisted of 50 mM sodium perchlorate in methanol/2-propanol (70/30, v/v) with the flow rate of 80 μL/min. The column oven was set to 40 °C. The oxidation potential for ECD was set to 650 mV. The level of reduced CoQ_9_ was expressed relatively as UbQH_2_/total CoQ ratio in each cell line.

### Statistical analysis

Unless specified otherwise, data are presented as mean value ± S.D. of at least three independent biological samples. For in vivo experiments, the number of mice per experimental group is stated in respective graphs (*n* ≥ 4). Statistical significance assessed using GraphPad Prism 7 software with *p* < 0.05 being regarded as significant is denoted as follows: ****p < 0.0001, ***p = 0.0001-0.001, **p = 0.001-0.01, and *p = 0.01–0.05. Two-tailed unpaired Student’s t-test and ANOVA (for multiple comparisons) were used where applicable. One-way ANOVA was adjusted with Dunnett’s multiple comparisons test, and two-way ANOVA was adjusted with Tukey’s multiple comparisons test. Curve comparison used to assess the statistical significance in survival experiments was done by Log-rank (Mantel-Cox) test. Contrast-adjusted images are representative of at least three independent experiments.

## Results

### Compromised respiration in glioblastoma cells delays tumour onset

**Fig. 1:**
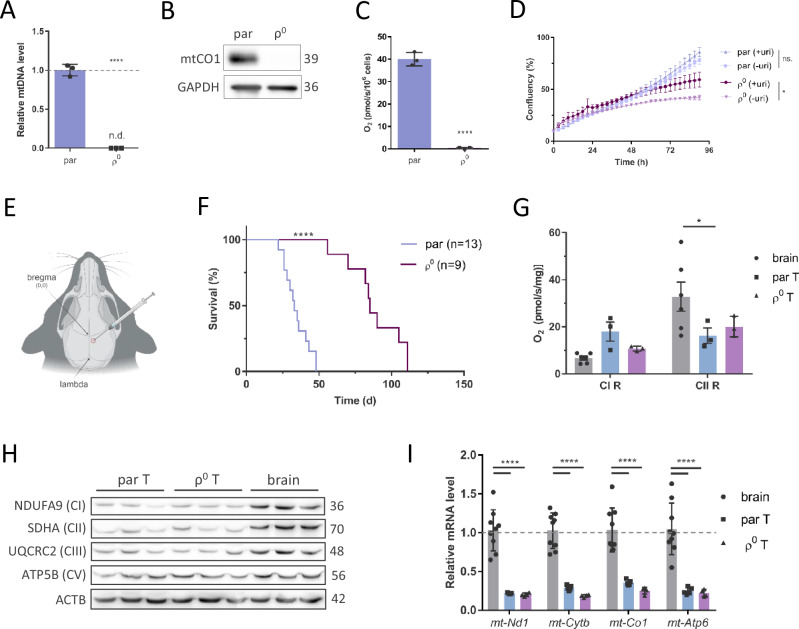
Glioblastoma cells without mtDNA form tumours with delay. Parental (par) and ρ^0^ GL261 cells were assessed for the level of mtDNA by qPCR (**A**), for the level of mtDNA-coded mtCO1 protein by WB (**B**), and for routine mitochondrial respiration using the Oxygraph (**C**). Dependency of ρ^0^ cells for exogenous uridine was confirmed by cultivation of parental and ρ^0^ cells in media with ( + uri) or without (-uri) added uridine (**D**). 5 × 10^4^ parental or ρ^0^ cells were implanted into the right hemisphere (coordinates: bregma 2,2) of syngeneic C57Bl/6 mice as indicated (**E**). Mice grafted with parental and ρ^0^ cells (*n* ≥ 9) were assessed for survival (**F**). Tumours formed from parental cells (par T) and from ρ^0^ cells (ρ^0^ T), and contralateral brain tissue were excised at the endpoint of the experiment (after detection of 15% animal weight loss and behavioural changes) and their CI- and CII-dependent respiration (CI R, CII R, respectively) was assessed by the Oxygraph (**G**), protein expression of subunits of respiratory complexes (CI, CII, CIII and CV) was assessed by WB (**H**), and gene expression of mtDNA-encoded genes by qRT-PCR (**I**).

To study mitochondrial transfer and the importance of functional mitochondrial ETC in brain tumours, we prepared respiration-deficient murine glioblastoma GL261 cells devoid of mtDNA (ρ^0^ cells) by long-term incubation in presence of low-dose ethidium bromide [20]. Depletion of mtDNA, evaluated by qPCR (Fig. 1A), led to the loss of protein expression of mtDNA-encoded genes, exemplified by mtCO1 (Fig. 1B), and resulted in aberrant respiration, assessed by high-resolution respirometry (Fig. 1C). GL261 ρ^0^ cells were cultured in media supplemented with pyruvate and uridine to support their proliferation (Fig. 1D). Next, we orthotopically implanted both parental (par) and ρ^0^ cells into the brain of immunocompetent syngeneic C57Bl/6 mice (Fig. 1E) to follow tumour progression, which was significantly delayed in the case of ρ^0^ cells, as determined by survival of animals within experimental ethical endpoint (Fig. 1F). In several animals, the ρ^0^ tumour growth was followed by MRI to confirm the tumour presence (Fig. S1A).

We excised the tumour and contralateral brain tissue at the endpoint of the survival experiments and evaluated respiration as well as protein and gene expression of mitochondrial respiratory complex subunits in homogenised tissues (Fig. 1G–I). We did not detect significant differences in respiration, mitochondrial gene expression or respiratory complex subunits between tumours formed from the parental (par T) and ρ^0^ cells (ρ^0^ T) at the endpoint, i.e. when the tumours were comparable in size, suggesting that mitochondrial function was restored in tumours derived from ρ^0^ cells. Since the time difference in tumour progression is significant, while the formed tumours were comparable in size and in several measured mitochondrial features at the endpoint, we hypothesised that prior to tumour onset from non-respiring ρ^0^ cells, these cells must adapt their mitochondrial metabolism, which may explain the observed tumour growth delay for ρ^0^ cells (Fig. 1F). Based on our previous results, we hypothesised that this may be due to the need for mitochondrial transfer from the surrounding tissue [13, 21], and we explored this concept further.

### ρ^0^ tumour cells acquire mitochondria from the host microenvironment in vivo

**Fig. 2:**
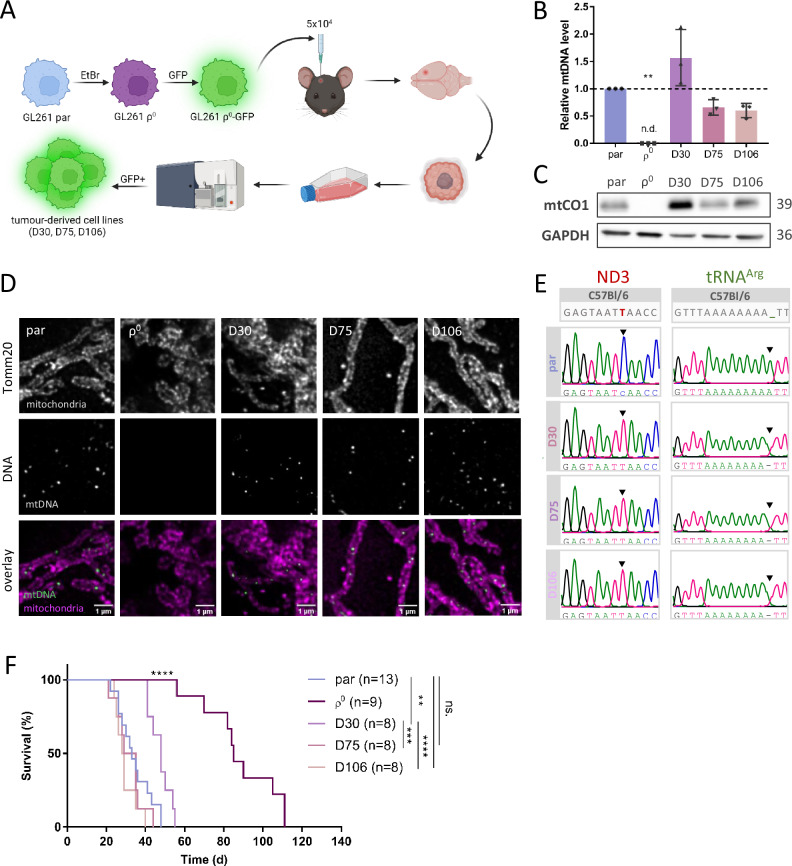
ρ^0^ cells acquire mitochondria with mtDNA from the host resulting in restored tumour growth. Tumour-derived cell lines D30, D75 and D106 were isolated after 30, 75 and 106 days post grafting, respectively, by sorting GFP-positive cells from tumours formed from GFP-tagged ρ^0^ cells implanted orthotopically into C57Bl/6 mice (**A**). The presence of mtDNA was assessed by qPCR (**B**) and visualised by stimulation emission depletion (STED) microscopy using anti-Tomm20 IgG to label mitochondrial membranes and anti-DNA IgG to label mitochondrial nucleoids; images were deconvolved and contrast-adjusted (**D**). Level of mtCO1 was probed for in tumour-derived cell lines by WB (**C**). The host identity of the mtDNA was confirmed by Sanger sequencing of two mtDNA polymorphic sites in *Nd3* and *tRNA*^*Arg*^ genes, with differences in polymorphic sites indicated by the black arrowhead (**E**). Tumour-derived cell lines were orthotopically injected into C57Bl/6 mice (*n* ≥ 8) to assess the animal survival, the ethical endpoint of the experiment being 15% animal weight loss and behavioural changes (**F**). EtBr – ethidium bromide.

To find whether HMT occurs in our model, we expressed GFP in the cytoplasm of GL261 ρ^0^ cells prior to engraftment into the brain to trace and sort cancer cells from tumours at different stages of growth for further analyses (Fig. 2A). We derived stable lines by fluorescence-activated cell sorting (FACS) of GFP-positive cells from excised tissues at various time points after grafting (30, 75 and 106 days). The D106 cells were established from a large endpoint tumour, while D30 and D75 cells were derived from the brain area around the injection site. The tumour used for the production of the D75 cell line was detectable by MRI but not visually, while mice did not have a detectable tumour 30 days after grafting (Fig. S1B). Therefore, D30 cells represent the pre-tumour stage, D75 the stage of tumour growth and D106 represents the large endpoint tumour stage. Evaluation of mtDNA content showed that while the original ρ^0^ cells do not contain any mtDNA, all of the ρ^0^ tumour-derived cells contain mtDNA (Fig 2B) and express mtDNA-encoded proteins, exemplified by mtCO1 (Fig. 2C). We supported these results by visualisation of the presence of DNA nucleoids in mitochondria by STED microscopy (Fig. 2D). We next confirmed the host origin of mtDNA in tumour-derived cell lines by sequencing parental mtDNA and mtDNA acquired by grafted GL261 ρ^0^ cells. We found that mtDNA of cells derived from tumours grown from ρ^0^ cells was of host origin and contained homoplasmic polymorphisms of host mtDNA in *Nd3* and *tRNA*^*Arg*^ genes (Fig. 2E). This presents unequivocal evidence of the host origin of mitochondria acquired by GL261 ρ^0^ cells with compromised respiration.

### Mitochondrial transfer to ρ^0^ tumour cells restores respiration and promotes tumour growth

We hypothesised that the HMT-linked acquisition of mtDNA by ρ^0^ cells restores tumour onset and growth. To test this, we orthotopically grafted parental, ρ^0^, D30, D75 and D106 cells in mice, and followed growth of syngeneic tumours. All tumour-derived cell lines with acquired mitochondria formed tumours significantly sooner than ρ^0^ cells. D75 and D106 cells formed tumours in a similar timespan as did parental cells, while D30 cells formed tumours with a short delay, probably reflecting the tumour stage and metabolic adaptations (Fig. 2F). Since this result points to the functional consequences of HMT in promoting tumour onset and progression, we investigated various mitochondrial characteristics linked to presence of mtDNA in more detail.

**Fig. 3:**
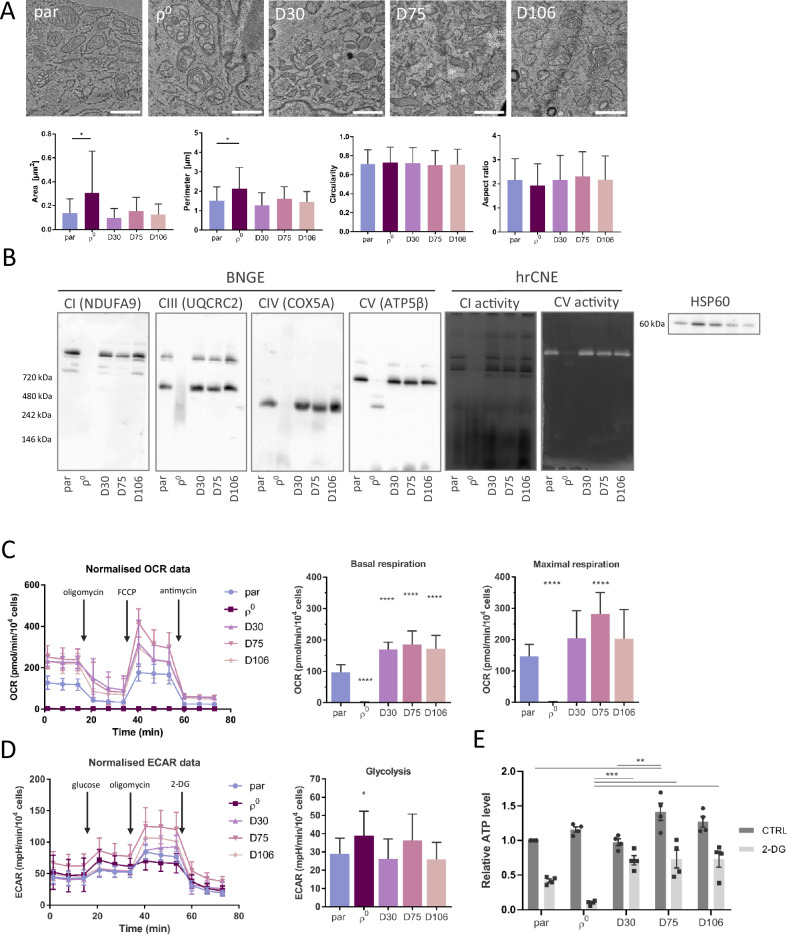
Mitochondria acquired by ρ^0^ cells in vivo recover mitochondrial cristae structure, assembly of respiratory complexes and respiration. Parental (par) and ρ^0^ cells, and cells derived from tumours grown from ρ^0^ cells at different times post grafting were assessed for mitochondrial morphology and cristae structure by transmission electron microscopy (TEM; **A**) and for assembly of respiratory complexes by blue native gel electrophoresis (BNGE) and their activity by high resolution clear native electrophoresis (hrCNE; **B**). Mitochondrial oxygen consumption rate (OCR; **C**) and extracellular acidification rate (ECAR; **D**) were evaluated using the Seahorse Extracellular Flux Analyzer. ATP level was evaluated using the CellTiter-Glo kit with 2-deoxyglucose (2-DG; 50 mM) used to inhibit glycolysis (**E**). FCCP - Carbonyl cyanide 4-(trifluoromethoxy) phenylhydrazone, CTRL – control.

Transmission electron microscopy (TEM) revealed that the ultrastructural morphology of mitochondria is disturbed in ρ^0^ cells, where mitochondria are larger in area and display cristae structure perturbances such as the onion-like morphology. Mitochondrial morphology in D30, D75 and D106 cells is similar to that in parental cells (Fig. 3A). Moreover, ρ^0^ cells display more fractionated mitochondrial networks than parental cells and the tumour-derived cell lines (Fig. S2A). We confirmed normalised expression of selected mtDNA-encoded genes in tumour-derived cell lines (Fig. S2B) and, linked to this, restored assembly of respiratory complexes (RCs) and supercomplexes (SCs), as determined by blue native gel electrophoresis (BNGE) as well as CI and CV in-gel activity (Fig. 3B). We also found that mitochondrial respiration recovered in tumour-derived cell lines compared to ρ^0^ cells (Fig. 3C) while ρ^0^ cells were highly glycolytic (Fig. 3D, E).

While mitochondrial content was elevated in ρ^0^ cells and all tumour-derived cells compared to parental cells (Fig. S2C), the mitochondrial membrane potential (ΔΨ_m_) and mitochondrial superoxide anion production in ρ^0^ cells was lower as would be expected in cells with dysfunctional ETC-dependent proton pumping (Fig. S2D, E). Our results confirmed that ρ^0^ cells maintain ΔΨ_m_, albeit lower than parental cells, due to the activity of sub-CV that pumps protons across the inner mitochondrial membrane (IMM) while converting ATP to ADP [34] (Fig. 3B). Conversely, ΔΨ_m_ in D30, D75 and D106 is elevated relative to the parental cells, indicating that the mitochondrial network in cells after HMT is hyperpolarised (Fig. S2D). Respiration and membrane potential recovery after mitochondrial transfer should allow for OXPHOS-derived ATP production. This raises the question of whether the reason for HMT in our model is a requirement for formation of ATP by OXPHOS in these GBM cells. We assessed ATP levels in the presence or absence of the glycolysis inhibitor 2-deoxyglucose (2-DG), and found that the total ATP level in ρ^0^ cells was comparable to the parental level in the absence of 2-DG and was negligible in the presence of the inhibitor. This points to bioenergetic plasticity of GBM cells, such that they utilise OXPHOS and glycolysis as needed depending on the microenvironment, indicating that ATP availability is not the limiting factor for tumour growth in GBM (Fig. 3E).

### Recovery of ETC by mitochondrial transfer reactivates DHODH function

**Fig. 4:**
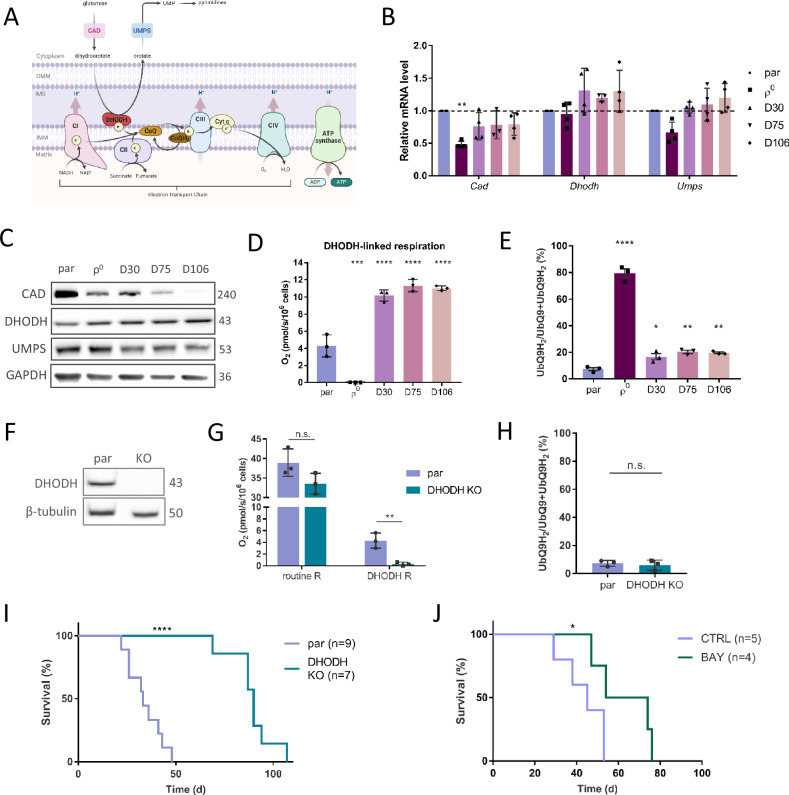
Respiration recovery by mitochondrial transfer propels DHODH-linked respiration. Graphical representation of de novo pyrimidine synthesis pathway converting glutamine to UMP and pyrimidines via the DHODH enzyme in cells with functional electron transport chain and CoQ redox-cycling. To drive forward the conversion od dihydroorotate to orotate, DHODH has to shuffle electrons to CoQ, whose redox-cycle is maintained by functional CIII and CIV (**A**). mRNA expression of the de novo pyrimidine synthesis pathway enzymes was measured by qRT-PCR (**B**) and protein expression was evaluated by WB (**C**) in parental and ρ^0^ cells, and in tumour-derived cell lines. DHODH-linked respiration was evaluated using the Oxygraph (**D**). The CoQ_9_ redox state was assessed by HPLC with electrochemical detection as a relative ratio of reduced/total CoQ_9_ (**E**). Parental and DHODH KO GL261 cells were assessed for the level of DHODH by WB (**F**), and routine and DHODH-linked respiration was evaluated by the Oxygraph (**G**). The CoQ_9_ redox state of DHODH KO cells was assessed by HPLC as a relative ratio of reduced/total CoQ_9_ (**H**). DHODH KO cells were orthotopically injected into mice and animal survival within the ethical endpoint was evaluated (**I**). Mice orthotopically injected with GL261 parental cells were treated with DHODH inhibitor BAY-2402234 daily by oral gavage until ethical endpoint (15% weight loss and behavioural changes) of the experiment was reached (**J**). CAD - carbamoyl-phosphate synthetase 2, aspartate transcarbamoylase, and dihydroorotase, DHODH – dihydroorotate dehydrogenase, UMPS - uridine monophosphate synthase, CoQ - ubiquinone, CoQH_2_ – ubiquinol, Cyt c – cytochrome c, UMP – uridine monophosphate, NAD(H) – nicotinamide adenine dinucleotide, OMM – outer mitochondrial membrane, IMM – inner mitochondrial membrane, IMS – intermembrane space, H^+^ - proton, e^-^ - electron, KO – knock-out, R – respiration, BAY - BAY-240223 treatment, CTRL – control, d - days.

Since we ruled out the requirement of OXPHOS as the source of ATP for GBM growth, we asked what other mitochondrial function necessary for GBM cell proliferation is aberrant in ρ^0^ cells. The finding that these cells proliferate less efficiently in media without uridine and pyruvate (Fig. 1D) indicates a dysfunctional de novo pyrimidine synthesis pathway that involves reactions converting glutamine to uridine 5-monophosphate (UMP) with the fourth step being catalysed by the mitochondrial enzyme DHODH (Fig. 4A). DHODH converts DHO to orotate, and the electrons generated are intercepted by the ETC component CoQ in its oxidised state. To maintain this step of the pathway, CoQ has to be redox-cycled, which is maintained by ETC activity. In ρ^0^ cells, the CoQ pool is largely composed of the reduced form of CoQ, UbQH_2_, that cannot drive de novo pyrimidine synthesis, as shown by evaluation of the UbQ/UbQH_2_ ratio [13]. On the other hand, the redox ratio of CoQ in parental and tumour-derived cell lines is shifted more to the oxidised state, capable of supporting DHODH function (Fig. 4E).

We found that even though the three proteins of the de novo pyrimidine synthesis pathway, the trifunctional CAD (carbamoyl-phosphate synthetase 2, aspartate transcarbamoylase, and dihydroorotase), DHODH and the bifunctional UMPS (uridine monophosphate synthase) are expressed on mRNA and protein level in all cell lines (Fig. 4B, C), DHODH-linked respiration is suppressed in ρ^0^ cells, and is restored in tumour-derived cells to an even higher level than in parental cells (Fig. 4D). This highlights the vital role of DHODH function in GBM growth. To confirm the crucial role of functional DHODH for GBM growth, we prepared GL261 DHODH knock-out (KO) cells (Fig. 4F) and validated the loss of DHODH-linked respiration (Fig. 4G). We also assessed UbQ/total CoQ ratio in this cell line and found it to be comparable with parental cells showing that the loss of DHODH enzyme does not interfere with CoQ redox cycling (Fig. 4H). Orthotopic grafting of parental and DHODH KO cells into mice revealed significantly delayed tumour formation by DHODH-deficient cells (Fig. 4I).

We next tested whether pharmacological inhibition of DHODH in vivo would affect tumour growth in a similar manner. We treated mice orthotopically grafted with parental cells with the blood-brain barrier penetrable DHODH inhibitor BAY-2402234 [35, 36] daily by oral gavage, starting one day after grafting. The treated group showed significantly longer survival than the control group (Fig. 4J). However, the tumour growth was not completely abrogated, and endpoint tumours were comparable in size. This suggests that proliferation of tumour cells subjected to metabolic alterations can be supported by other components of the tumour microenvironment in vivo [37]. Overall, these results corroborate recent studies that point to the potential therapeutic benefits of DHODH inhibition in brain cancer [38, 39].

### Metabolic tracing reveals the importance of a functional ETC for pyrimidine production

**Fig. 5:**
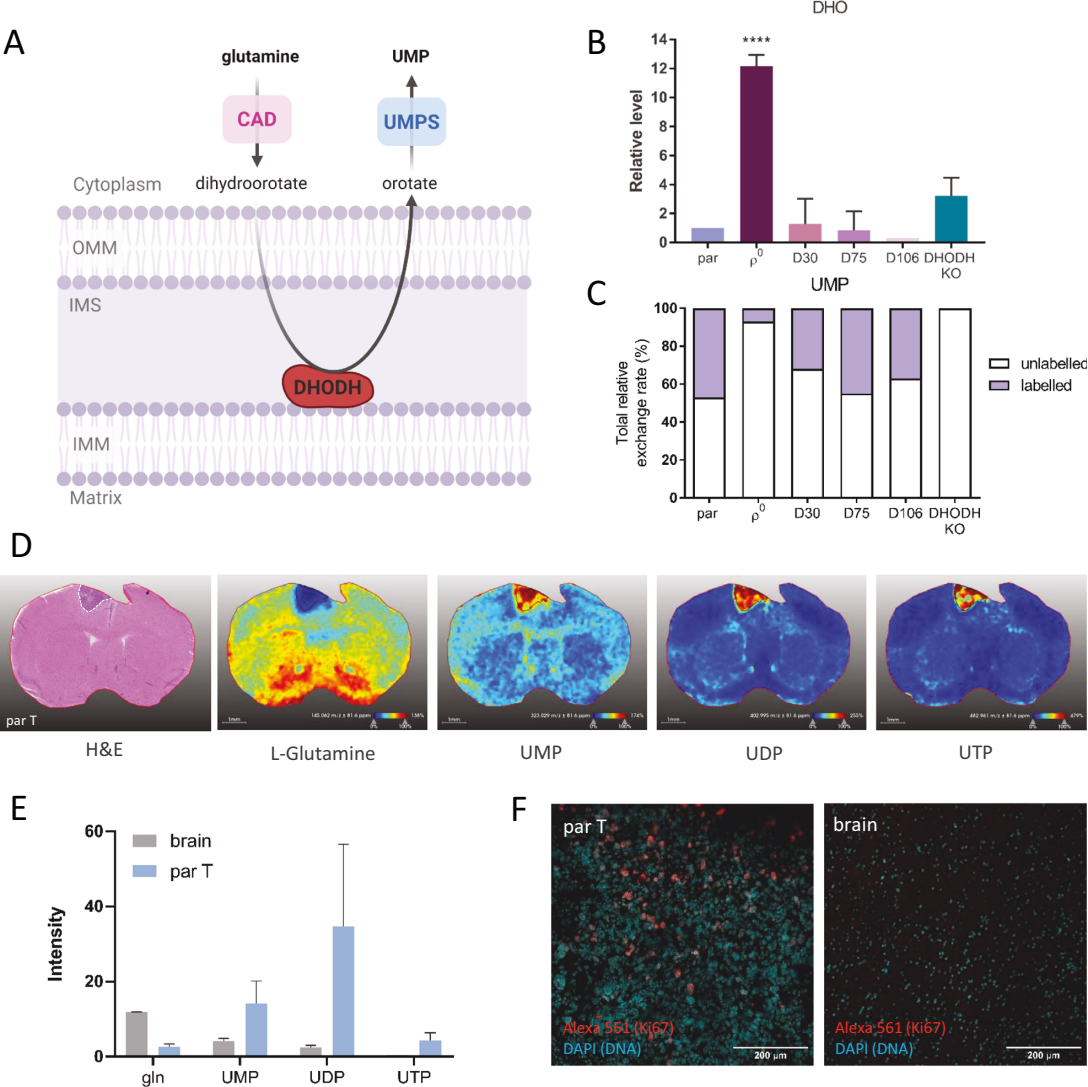
DHODH plays an important role in tumour growth. Levels of metabolites of de novo pyrimidine synthesis pathway were determined by LC-MS/MS (**A**). Total level of DHO was normalised to protein content and expressed relative to parental cells (**B**). Stable isotope tracing of labelled glutamine was used to determine the efficiency of the de novo pyrimidine synthesis pathway and the levels of labelled UMP were normalised to protien and expressed as total relative exchange rate in each cell line (**C**). Levels of de novo pyrimidine synthesis pathway metabolites in the brain and parental tumour (par T) tissues, visualised by H&E staining, were detected by MALDI (**D**) and mean intensities were plotted into a graph (*n* = 2; **E**). Sections of brains bearing par tumours (par T) were stained against proliferation marker Ki67 and counterstained with DAPI to visualise DNA (**F**). OMM – outer mitochondrial membrane, IMM – inner mitochondrial membrane, IMS – intermembrane space, CAD - carbamoyl-phosphate synthetase 2, aspartate transcarbamoylase, and dihydroorotase, DHODH - dihydroorotate dehydrogenase, UMPS - uridine monophosphate synthase, UMP – uridine monophosphate, UDP - uridine diphosphate, UTP - uridine triphosphate, H&E – hematoxylin and eosin.

To demonstrate the necessity of functional ETC for de novo pyrimidine synthesis we assessed the levels of the pathway’s metabolites (Fig. 5A). In ρ^0^ cells with compromised ETC and in DHODH KO cells, we detected the overall accumulation of DHO, which is the metabolite in de novo pyrimidine synthesis pathway directly preceding the enzymatic conversion by DHODH enzyme (Fig. 5B). This confirms that dysfunctional ETC disables the enzymatic conversion of DHO to orotate via DHODH. To determine the origin of UMP present in the cell lines, we performed stable isotope tracing of labelled glutamine in the cells after 24 h incubation with this compound. In accordace with the DHO accumulation, ρ^0^ cells and DHODH KO cells contain almost none labelled UMP that comes from the de novo pyrimidine synthesis pathway (Fig. 5C). On the other hand, labelled UMP was detected in parental cells and all ρ^0^ tumour-derived cell lines.

GBM is highly proliferative tissue compared to the surrounding healthy brain regions (Fig. 5F) and therefore expected to be more dependable on pyrimidine de novo synthesis. To compare the level of metabolites present in healthy brain or tumour tissue, we performed MALDI imaging of samples from mice bearing tumours formed from parental cells (Fig. 5D). We observed that while glutamine is ubiquitously present in the brain tissue, it is completely absent in the tumour area. On the other hand, tumour tissue compared to brain tissue is highly enriched in downstream metabolites of de novo pyrimidine synthesis pathway, UMP, UDP and UTP (Fig. 5D, E), pointing to the high dependency on de novo pyrimidine synthesis in GBM.

### Astrocytes are the most prominent mitochondrial donor cell type within the brain

**Fig. 6:**
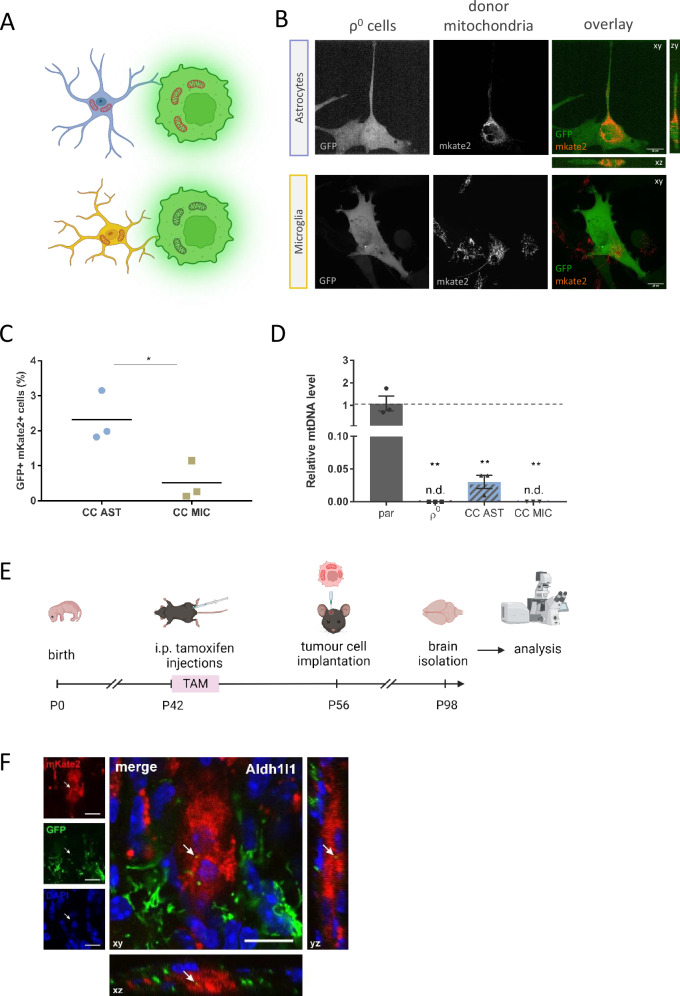
Astrocytes are the predominant mitochondrial donors for ρ^0^ GBM cells. To determine which brain resident cell type is the donor of mitochondria for cancer cells, in vitro co-culture experiments with two cell types isolated from adult mouse brain were carried out (**A**). Co-cultures of neural cells and GL261 ρ^0^ GFP^+^ cells were assessed by fluorescent confocal microscopy after 48 h (**B**) and by flow cytometry after 24 h (**C**). mtDNA levels were detected in parental and ρ^0^ cells, and in ρ^0^ cells after their 72-h co-culture with potential neural donor cells (**D**). Horizontal transfer of mitochondria from astrocytes in vivo was confirmed by confocal microscopy (**F**) using the inducible MitoTag mice model as visualised (**E**). CC AST – co-culture with astrocytes, CC MIC - co-culture with microglia, TAM – tamoxifen, i.p. – intraperitoneal, P – postnatal day, Aldh1l1 – aldehyde dehydrogenase 1l1.

We showed that GL261 ρ^0^ cells obtain mitochondria from cells in the host microenvironment, which led us to investigate the identity of mitochondrial donors, employing first in vitro co-culture models (Fig. 6A). We isolated two neural cell types, astrocytes and microglia, from the brains of adult mito::mKate2 mice with mitochondria expressing the far red fluorophore, mKate2. Magnetic bead separation was based on the expression of specific markers (astrocytes: ACSA-2, microglia: CD11b), confirmed by antibody staining against GFAP (glial fibrillary acidic protein) and IBA1 (ionised calcium-binding adaptor molecule 1) respectively (Fig. S3A). We also assessed the localisation of these cell types in brain sections from ρ^0^ tumours and found that they both localise around the tumour border and that microglia also infiltrate the tumour area (Fig. S3B). Using confocal microscopy, we observed mitochondrial transfer to GL261 ρ^0^-GFP cells from astrocytes, while no observable transfer from microglia occured within 48 h (Fig. 6B). We quantified mitochondrial transfer by flow cytometry in co-cultures after 24 h and found that GL261 ρ^0^-GFP cells obtained mitochondria from astrocytes with higher frequency than from microglia (Fig. 6C, Fig. S3C). qPCR evaluation of mtDNA content in GL261 ρ^0^-GFP cells after 72 h co-culture confirmed these results, i.e. that mtDNA was present in GBM cells after co-culture with astrocytes but was not detected in co-cultures with microglia (Fig. 6D).

To show that astrocytes act as mitochondrial donors for brain tumours in vivo, we prepared MitoTag mice with inducible astrocyte-specific mitochondrial GFP expression (Fig. S3D, E) and injected them orthotopically with GL261 ρ^0^-mito:mKate2 cells expressing the far red fluorophore mKate2 in mitochondria. After six weeks, brains were excised, sectioned around the injection site, and evaluated by confocal microscopy (Fig. 6E). We detected the transfer of green mitochondria into the grafted mito:mKate2 cells in Aldh1l1:MitoTag mice, showing that astrocytes act as donors of mitochondria to GBM cells with compromised respiration in vivo, allowing for brain tumour onset and growth (Fig. 6F). We did not observe similar mitochondrial transfer from microglial cells in vivo.

## Discussion

Horizontal mitochondrial transfer is now a widely recognised process in the context of tumour formation in various cancer types [20, 40–42]. Recent publications highlight the pivotal role of mitochondrial metabolism in tumour formation and growth, which may be seemingly at odds with the Warburg effect and the notion that aerobic glycolysis is the main pathway to fuel cancer growth [14, 43]. It has been shown that OXPHOS deficiencies and ETC drug targeting restrain tumour growth, placing mitochondria into the cancer research spotlight [44–46]. We have shown previously that cancer cells with respiration deficiency caused by mtDNA depletion could only form tumours after mitochondrial acquisition from the host tumour microenvironment by HMT, i.e. by acquisition of mtDNA by means of intercellular movement of whole mitochondria [20, 21]. In case of GBM, HMT has been linked to increased proliferative capacity, self-renewal or resistance to treatment [28, 47, 48]. In the current study, we used a model of respiration-deficient GBM cells to investigate possible HMT requirement for growth of this brain tumour type. We employed an orthotopic mouse model to study this phenomenon in the native microenvironment where GBM cells interact with non-malignant cells of the tumour stroma. Since ρ^0^ cells do not contain mtDNA, we could confirm the occurrence of HMT by detecting mtDNA in tumour cells that grew from GL261 ρ^0^ cells, sorted from tumours at different time points. Another advantage of our model of inbred mice was that it allowed for unequivocal documentation of the host origin of mtDNA, based on homoplasmic mtDNA polymorphisms between the original GL261 cells and C57Bl/6 mice from which they were derived. Lastly, the ρ^0^ GBM model allowed us to confirm the requirement for a functional ETC for brain tumour progression.

In our previous work on HMT in tumour formation, we used subcutaneous models of breast cancer and melanoma ρ^0^ cells [20, 21], where the lag period before tumour appearance was 2–3 weeks. In the present orthotopic GBM model, the median survival of mice with parental tumours was 33 days, while the survival of mice with tumours derived from ρ^0^ cells was 85 days, representing a 2-month delay. We found that mtDNA of host origin was present in ρ^0^ tumour-derived cell lines as early as 30 days after grafting (D30 cells). This corroborates our previous findings from subcutaneous models that HMT between stroma and respiration-deficient cancer cells is important in the initial stages of tumour growth and argues that for ρ^0^ cells, this interaction is crucial for in vivo tumour onset and growth. Indeed, all cell lines isolated from tumours that grew from ρ^0^ cells formed tumours in vivo more readily than ρ^0^ cells. Cell lines derived from MRI-detectable tumours (D75 and D106) did not exhibit any lag period in tumour growth compared to parental cells, demonstrating that this adaptation, i.e. acquisition of functional mitochondria, was sufficient to restore the tumorigenic capacity of the cells. The fact, that D30 cells, which had already acquired host mitochondria, grow tumours with a slight delay compared to parental, D75 and D106 cells, cannot be explained by the differences in HMT, but rather by the difference in stage of the tumour growth when the cells were derived, i.e. at the early stage when the tumour was not yet visible. The delay in tumour growth by D30 cells likely reflects the state of the cells in the original tumour where additional metabolic adaptations are still required, even though HMT has already occurred and DHODH activity is restored. In fact, D30 cells have higher mitochondrial and mtDNA content than parental cells and produce most of their ATP by means of OXPHOS rather than glycolysis. Together, these results show that HMT is an important, early event preceding growth of GBM from cancer cells lacking a functional ETC.

Cells that acquired mitochondria from the host tissue have restored mitochondrial supercomplex assembly, mitochondrial respiration and OXPHOS-linked ATP production. Therefore, we assessed ATP formation in more detail and found that ρ^0^ cells produce ATP at levels comparable to parental cells, although this process is maintained in ρ^0^ cells by glycolysis. This supports previous observations that OXPHOS-linked ATP production is not essential for tumour growth [13, 16, 49, 50] and the notion that glycolysis is kinetically superior to OXPHOS [51]. On the other hand, mtDNA deficiency in ρ^0^ cells leads to impaired CIII/CIV SC assembly, as shown by BNGE, causing perturbations in CoQ redox cycling and accumulation of reduced CoQ, UbQH_2_. Oxidised CoQ, UbQ, drives de novo pyrimidine synthesis by means of the mitochondrial enzyme DHODH, which converts DHO to orotate. Recently, it was shown that defects in CIII abrogate tumour growth and that ubiquinol oxidation is necessary for tumour growth [13, 14, 52]. Even though DHODH protein expression is unchanged in ρ^0^ cells, these cannot perform DHODH-linked respiration because CoQ redox cycling is impaired. On the contrary, ρ^0^ tumour-derived cell lines that acquired mitochondria via HMT are capable of UbQH_2_ re-oxidation and DHODH-linked respiration with subsequent UMP formation. Stable isotope tracing of labelled glutamine showed that ρ^0^ and DHODH KO cells are not able to use glutamine for de novo pyrimidine synthesis. In contrast, ρ^0^ tumour-derived cell lines after mitochondrial acquisition produced UMP levels comparable to parental cells, in line with our previous results with solid subcutaneous tumours [13]. Moreover, the highest level of labelled UMP apart from parental cells was detected in D75 cell line that represents the tumour growth stage that most likely has the highest need for pyrimidine production for proliferation.

We also show here that in highly proliferative tumour tissue compared with brain tissue, glutamine is undetectable, most likely because it is used to support de novo pyrimidine synthesis, since in the same area, the production of UMP is highly elevated. This points to a substantial need for de novo pyrimidine synthesis in glioblastoma growth in vivo and explains the importance of HMT prior to detectable tumour onset. The central role of DHODH in glioblastoma growth is corroborated by the fact that GL261 DHODH KO cells that lack DHODH-linked respiration but otherwise display functional OXPHOS formed tumours with considerable delay. Moreover, the blood-brain-barrier penetrable DHODH inhibitor BAY-2402234 prolonged survival of treated mice compared to the control group. The eventual growth of tumours in the treated group could also have been supported by other components of the tumour microenvironment. Overall, our results point to the essential role of a functional ETC in glioblastoma growth, as ETC deficiency in ρ^0^ cells precludes tumour growth, and acquisition of functional mitochondria is its prerequisite. Mechanistically, a functional ETC is required to maintain CoQ redox cycling and drive the de novo pyrimidine synthesis via DHODH, while ATP production supported by OXPHOS is dispensable for GBM onset and growth.

To obtain a more comprehensive understanding of the role of HMT in brain cancer, we explored which brain cell types act as mitochondrial donors for ρ^0^ cells. Using an in vitro approach, we showed that astrocytes are the most prominent donors of mitochondria, which is in line with a recent study [28]. Moreover, astrocytes have also been implicated in mitochondrial transfer in the opposite direction, where cancer cells transfer tumour cell-derived mitochondria to adapt astrocytes to tumour-like metabolism [53, 54]. These findings point to the possibility of the occurrence of bidirectional mitochondrial transfer, which would benefit cancer cells in two ways: promoting their tumorigenicity and adapting the microenvironment to better suit their metabolic needs. Contrary to the recent paper concerning HMT in GBM [28], we could not detect any substantial mitochondrial transfer from microglia by confocal microscopy in our experimental model. The results of these co-culture experiments also clearly show that mitochondrial functions are restored due to the process of HMT rather than mtDNA transfer alone, in accordance with previous studies [21, 55].

To show the role of astrocytes as donors of mitochondria for tumour growth in vivo, we prepared transgenic MitoTag mice with inducible astrocyte-specific mitochondrially targeted expression of GFP and injected them with GL261 ρ^0^-mito:mKate2 cells expressing a far red fluorophore in mitochondria. Brains were examined by confocal microscopy six weeks after cell injection to evaluate mitochondrial transfer at early stages, prior to discernible tumour appearance when HMT occurrence can be expected. We observed HMT in vivo, albeit at low level, based on the presence of GFP-positive astrocyte mitochondria inside mito:mKate2-positive mitochondrial network of acceptor cancer cells, qualitatively showing that astrocytes can transfer mitochondria to cancer cells in vivo. The low frequency of HMT from astrocytes to GL261 ρ^0^ cells in vivo may be due to several reasons. One is that transfer of mitochondria in vivo is a rather rare event, the other is that the mitochondrial fluorescent label of the donor cells will fade soon after the transfer, as the transgene encoding the fluorescent protein remains with the host donor cell. Concerning the latter, we found very low apparent frequency of mitochondrial transfer from host stroma into subcutaneously grafted B16 ρ^0^ cells, such that we detected only 0.52% of double positive cells [21]. On the other hand, the low frequency of mitochondrial transfer from the stroma to respiration-deprived GBM cells will suffice to repopulate GL261 ρ^0^ cells with functional mitochondria to support brain tumour growth.

In conclusion, our observation that respiration-deficient ρ^0^ GBM cells cannot form tumours unless they acquire functional mitochondria from the host microenvironment led to the conclusion that functional mitochondrial respiration is essential for GBM onset and progression via support of CoQ redox cycling that drives DHODH-dependent de novo pyrimidine synthesis. These results provide important insights into GBM growth and corroborate several recent studies reporting similar mechanisms in different cancer models [13, 14]. Moreover, this work supports possible 2-pronged therapeutic intervention for GBM treatment, either by inhibition of DHODH or inhibition of mitochondrial transfer [36, 56]. It cannot be ruled out that HMT during GBM treatment can account for at least part of the resistance of this aggressive cancer [47]. Although inhibition of mitochondrial transfer alone may not be beneficial in later stages of cancer progression, there have been efforts to target HMT for cancer treatment [56, 57]. Since HMT likely promotes chemoresistance by increasing the mitochondrial fitness in GBM cells, targeting intercellular movement of mitochondria in combination with other anti-cancer approaches may provide benefit to GBM patients.

## Supplementary information

Supplementary tables

Supplementary data

## Data Availability

The datasets generated during and/or analysed during the current study are available from the corresponding author J. Neuzil on reasonable request.
