# Correction: Functional mitochondrial respiration is essential for glioblastoma tumour growth

**DOI:** 10.1038/s41388-025-03451-8

**Published:** 2025-06-04

**Authors:** Petra Brisudova, Dana Stojanovic, Jaromir Novak, Zuzana Nahacka, Gabriela Lopes Oliveira, Ondrej Vanatko, Sarka Dvorakova, Berwini Endaya, Jaroslav Truksa, Monika Kubiskova, Alice Foltynova, Daniel Jirak, Natalia Jirat-Ziolkowska, Lukas Kucera, Karel Chalupsky, Krystof Klima, Jan Prochazka, Radislav Sedlacek, Francesco Mengarelli, Patrick Orlando, Luca Tiano, Paulo Oliveira, Carole Grasso, Michael V. Berridge, Renata Zobalova, Miroslava Anderova, Jiri Neuzil

**Affiliations:** 1 Institute of Biotechnology, Czech Academy of Sciences, 252 50 Vestec, Czech Republic; 2 Faculty of Science, Charles University, 128 00 Prague 2, Czech Republic; 3 CNC-UC, Center for Neuroscience and Cell Biology, University of Coimbra, 3060-197 Cantanhede, Portugal; 4 CCIBB, Centre for Innovative Biomedicine and Biotechnology, University of Coimbra, 3060-197 Cantanhede, Portugal; 5 PPDBEB, Institute for Interdisciplinary Research, Doctoral Programme in Experimental Biology and Biomedicine, University of Coimbra, 3060-197 Cantanhede, Portugal; 6 Institute of Experimental Medicine, Czech Academy of Sciences, 142 00 Prague 4, Czech Republic; 7 Second Faculty of Medicine, Charles University, 150 06 Prague 5, Czech Republic; 8 Institute of Clinical and Experimental Medicine, 140 21 Prague 4, Czech Republic; 9 Institute of Biophysics and Informatics, First Faculty of Medicine, Charles University, 121 08 Prague 4, Czech Republic; 10 Czech Centre for Phenogenomics, Institute of Molecular Genetics, Czech Academy of Sciences, 142 20 Prague 4, Czech Republic; 11 Department of Life and Environmental Sciences, Polytechnic University of Marche, 60131 Ancona, Italy; 12 Malaghan Institute of Medical Research, Wellington, 6242 New Zealand; 13 First Faculty of Medicine, Charles University, 121 08 Prague 2, Czech Republic; 14 School of Pharmacy and Medical Science, Griffith University, Southport, QLD 4222 Australia

**Keywords:** CNS cancer, Cell growth

Correction to: *Oncogene* 10.1038/s41388-025-03429-6, published online 5 May 2025

In this article, the author’s name Paulo J. Oliveira was incorrectly written as Paulo Oliveira. The original article has been corrected.

