# Supplementary material for: Functional mitochondrial respiration is essential for glioblastoma tumour growth
Source: Oncogene. 2025 May 5;44(30):2588–603. doi: 10.1038/s41388-025-03429-6 (PMC12277175; doi:10.1038/s41388-025-03429-6)
Supplement: Supplementary file 1 — Supplementary tables [file 41388_2025_3429_MOESM1_ESM.docx]

**Supplementary Table 1:** Primer sequences

|  | **Gene** | **Forward sequence** | **Reverse sequence** |
| --- | --- | --- | --- |
| qRT-PCR | *Actb* | CATTGCTGACAGGATGCAGAAGG | TGCTGGAAGGTGGACAGTGAGG |
|  | *Rn18s* | CGCCGCTAGAGGTGAAATTCT | CGAACCTCCGACTTTCGTTCT |
|  | *Mito1* | CTAGAAACCCCGAAACCAAA | CCAGCTATCACCAAGCTCGT |
|  | *mt-Nd1* | CAGCCGGCCCATTGGCGTTA | AGCGGAAGCGTGGATAGGATGC |
|  | *mt-Cytb* | TCCTTCATGTCGGACGAGGC | AATGCTGTGGCTATGACTGCG |
|  | *mt-Co1* | TCAACATGAAACCCCCAGCCA | GCGGCTAGCACTGGTAGTGA |
|  | *mt-Atp6* | AGCTCACTTGCCCACTTCCT | AAGCCGGACTGCTAATGCCA |
|  | *Cad* | TACGCAGTTCTCATCGACCA | TGGGAGTTGCATGAAGAGTG |
|  | *Dhodh* | TGAGGAGCCTACAGGGAAAGAC | ACGCTGGCAATGTCCTCCTTGT |
|  | *Umps* | TGGCGACAGTTATCTGCTCAGC | CGTCCTCAATGACCAGACAGGT |
| sequencing | *mtDNA1* | TGTCCTGATCAATTCTAGTAGTTCC | CTTTATTGGTGGCTGCTTTTAGGC |
|  | *mtDNA2* | TTCAATTTTAAACTTGCTAA | TTGTTTCTGCTAGGGTTGAG |
|  | *mtDNA3* | TATGAAGTAACCATAGCTATTATCC | AGTCCTTATCAGAAGTTAAACTTGT |
|  | *mtDNA4* | AACCCAACCTAATATTTTCCACCCT | ATGATGAGAACAGCTGTTAGTGAAA |
|  | *mtDNA5* | GCCATCATATTCGTAGGAGTAAACA | CCTGCAGTAATGTTAGCTGTAAGCC |
|  | *mtDNA6* | CAGGCTTCCGACACAAACTAAAAA | TTTGAAACTTTTACTAGTAGGGCTA |
|  | *mtDNA7* | ATGCCTGGAAGGCATAGTATTATCC | TGTGTTTAATTAGGTTTGATATTGAAA |
|  | *mtDNA8* | ACTTTTATAGGATAATAGTAATC | GATGATGTTAATTAGGAATGATATAAAGTA |
|  | *mtDNA9* | AGCCTAAAAACATCCCTAACTCTCC | TGATTGAGAAGTATGAGATGGAGGC |
|  | *mtDNA10* | ACCTCAAAGCAACGAAGCCTAATA | GAACAGGCTCCTCTAGATGGATATA |

|  | **Antibody** | **Source** | **Identifier** |
| --- | --- | --- | --- |
| WB | anti-NDUFA9 | Abcam | ab14713 |
|  | anti-SDHA | Abcam | ab137040 |
|  | anti-UQCRC2 | Abcam | ab14745 |
|  | anti-ATP5B | Sigma-Aldrich | HPA001520 |
|  | anti-CAD | Cell Signalling | #93925 |
|  | anti-DHODH | Proteintech | 14877-1-AP |
|  | anti-UMPS | Santa Cruz | sc-398086 |
|  | anti-MTCO1 | Abcam | ab14705 |
|  | anti-GFP | Invitrogen | A-11122 |
|  | anti-ACTB | Cell Signalling | #8457 |
|  | anti-GAPDH | Cell Signalling | #5174 |
|  | anti-β-Tubulin | Cell Signalling | #86298 |
| BNGE | anti-NDUFA9 | Abcam | ab14713 |
|  | anti-UQCRC2 | Abcam | ab14745 |
|  | anti-COX5A | Abcam | ab110262 |
|  | anti-ATP5B | Sigma-Aldrich | HPA001520 |
|  | anti-HSP60 | Cell Signalling | #12165 |
| IF | anti-GFAP | Abcam | ab7260 |
|  | anti-IBA1 | Abcam | ab178847 |
|  | anti-Ki67 | Cell Signalling | #9027 |
| secondary antibodies | Goat Anti-Mouse IgG (H+L)-HRP Conjugate | Biorad | #1706516 |
|  | Goat Anti-Rabbit IgG (H+L)-HRP Conjugate | Biorad | #1706515 |
|  | Alexa Fluor™ 488, Goat anti-Rabbit IgG (H+L) | Invitrogen | A-11008 |
|  | Alexa Fluor™ 568, Goat anti-Rabbit IgG (H+L) | Invitrogen | A-11036 |
| STED | anti-DNA | Progen | 61014 |
|  | anti-TOMM20 | Abcam | ab186735 |
|  | abberior STAR RED, goat anti-mouse IgG | abberior | STRED-1001-500UG |
|  | abberior STAR 580, goat anti-rabbit IgG | abberior | ST580-1002-500UG |

**Supplementary Table 2:** Antibody list
