# Supplementary material for: Functional mitochondrial respiration is essential for glioblastoma tumour growth
Source: Oncogene. 2025 May 5;44(30):2588–603. doi: 10.1038/s41388-025-03429-6 (PMC12277175; doi:10.1038/s41388-025-03429-6)
Supplement: Supplementary file 2 — Supplementary data [file 41388_2025_3429_MOESM2_ESM.pdf]

Supplementary Figure 1

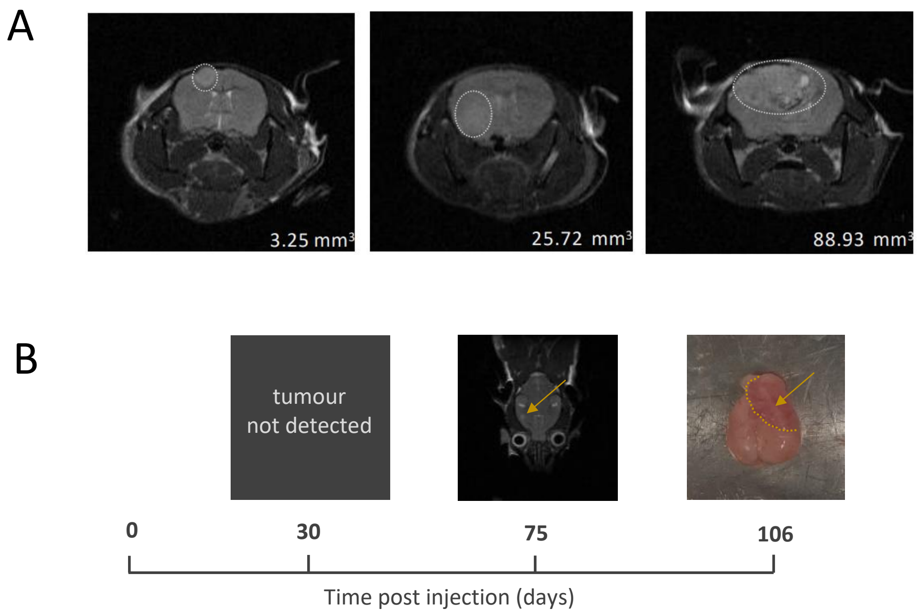

Supplementary Figure 1

Tumour presence in mice was confirmed by magnetic resonance imaging (MRI; A). Tumour-derived cell lines D75 and D106 were prepared from tumours detected by MRI or visually, while D30 cell line was prepared from tissue surrounding the injection site from a mouse that did not bear detectable tumour (B).

Supplementary Figure 2

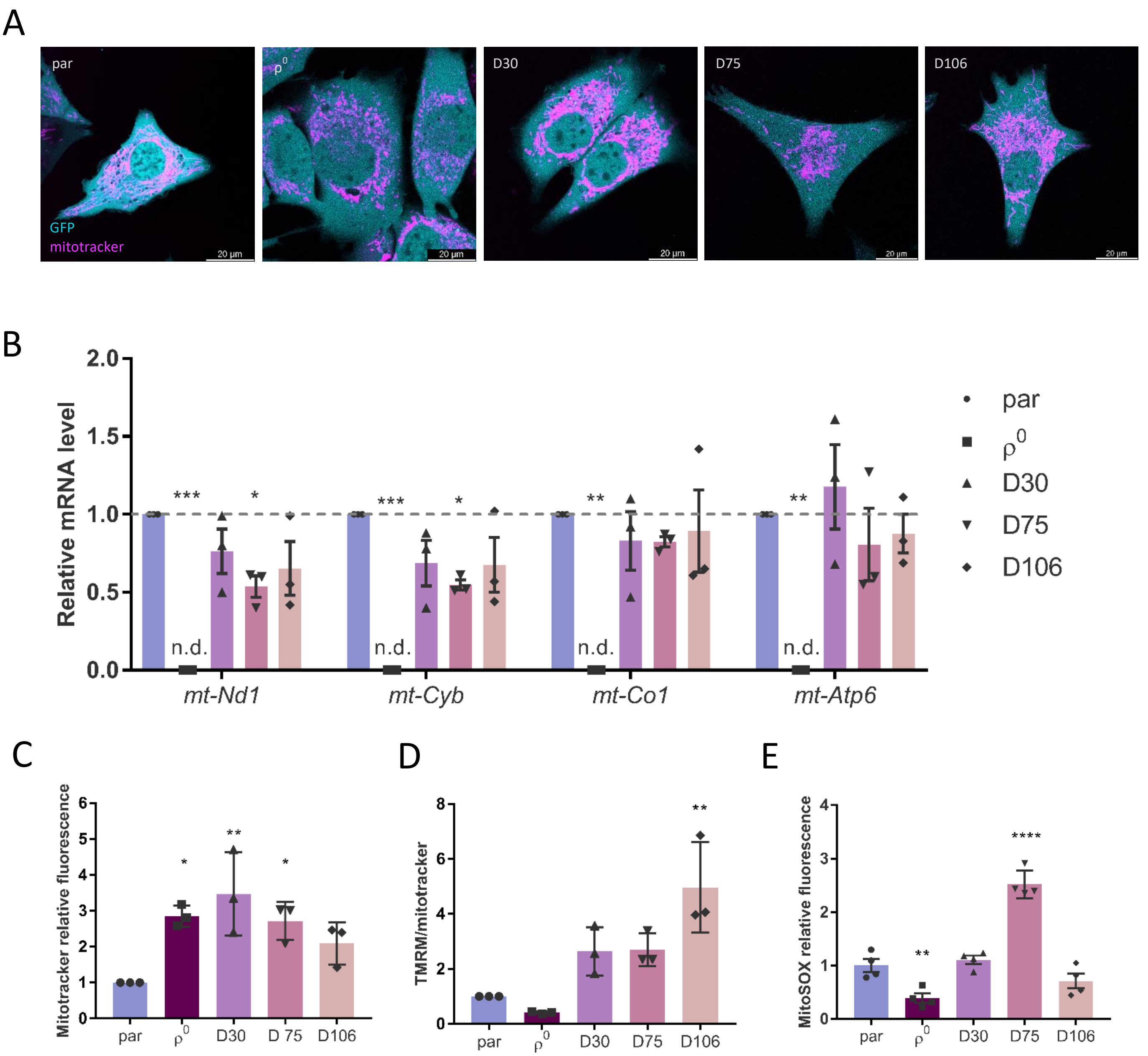

Supplementary Figure 2

Mitochondrial network of tumour-derived cell lines stained with MitoTracker was visualised by live confocal fluorescent microscopy (A). Level of several mtDNA-coded genes was evaluated in tumour-derived cell lines by qRT-PCR (B). Mitochondrial amount (C), membrane potential (D) and ROS levels (E) were assessed by flow cytometry. TMRM - Tetramethylrhodamine, methyl ester.

Supplementary Figure 3

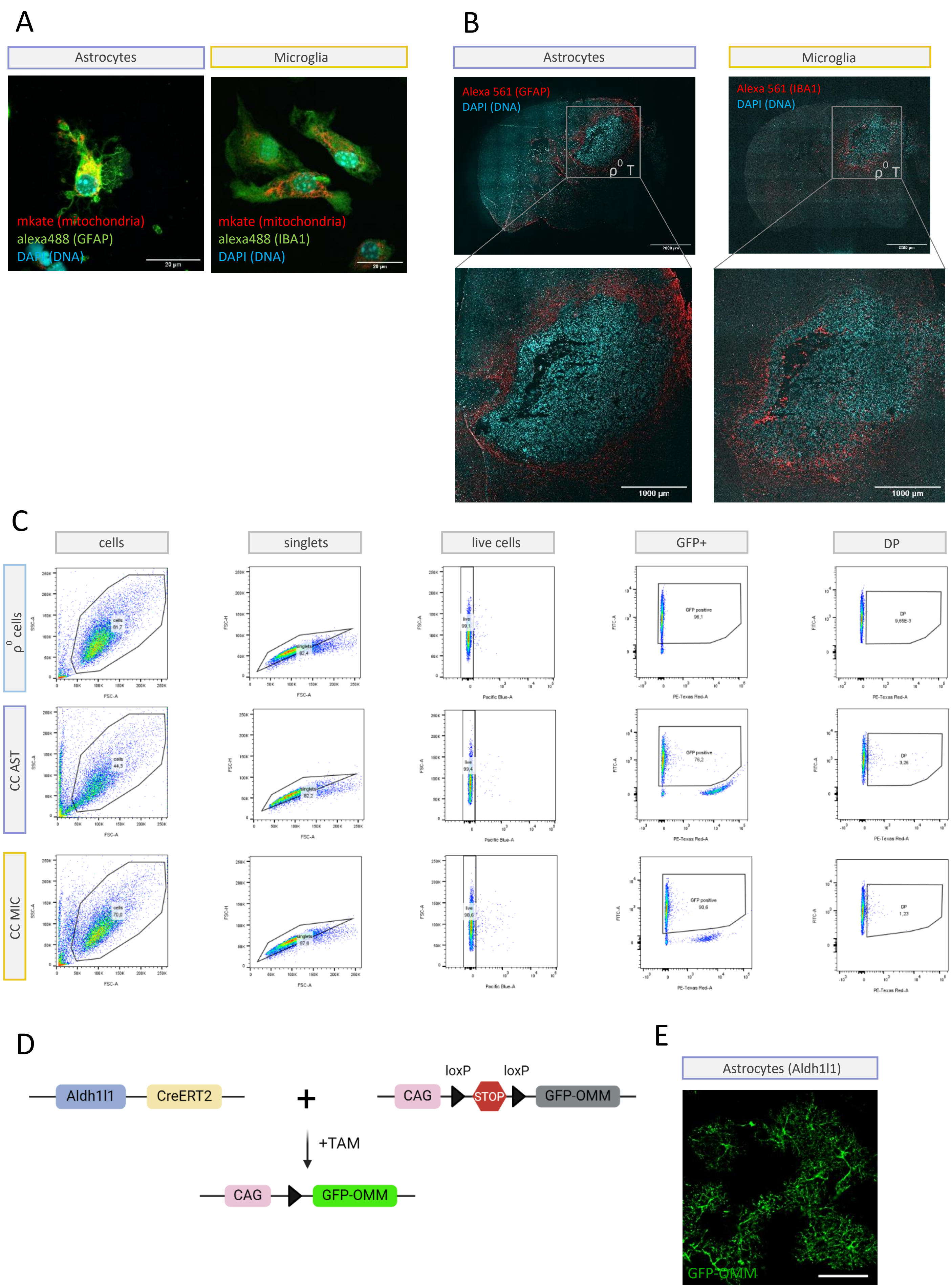

Supplementary Figure 3

Neural cells isolated from adult mouse brains were labelled with antibodies against their specific markers and visualised by confocal fluorescent microscopy (A). Brains of mice bearing ρ^0 tumours (ρ^0 T) were sectioned and stained with antibodies against markers of specific neural cell types (B). Flowcytometry gating strategy of co-culture experiments (C). Visual representation of mouse model breeding for the creation of cell-specific expression of mitochondrially located GFP (D). Microscopy images of mitochondrial network in astrocytes and microglia of tamoxifen induced MitoTag mouse brain (E). GFAP – Glial fibrillary acidic protein, IBA1 - Ionized calcium binding adaptor molecule 1, CC AST - co-culture with astrocytes, CC MIC - co-culture with microglia, FSC-A – forward scatter area, SSC-A – side scatter area, FSC-H – forward scatter height, DP – double positive, Aldh1l1 – aldehyde dehydrogenase 1l1, TAM – tamoxifen, GFP-OMM – green fluorescent protein tagged outer mitochondrial membrane.
